# Glucose-6-P/phosphate translocator2 mediates the phosphoglucose-isomerase1-independent response to microbial volatiles

**DOI:** 10.1093/plphys/kiac433

**Published:** 2022-09-16

**Authors:** Samuel Gámez-Arcas, Francisco José Muñoz, Adriana Ricarte-Bermejo, Ángela María Sánchez-López, Marouane Baslam, Edurne Baroja-Fernández, Abdellatif Bahaji, Goizeder Almagro, Nuria De Diego, Karel Doležal, Ondřej Novák, Jesús Leal-López, Rafael Jorge León Morcillo, Araceli G Castillo, Javier Pozueta-Romero

**Affiliations:** Instituto de Agrobiotecnología (IdAB), CSIC-Gobierno de Navarra, Iruñako etorbidea 123, 31192 Mutiloabeti, Nafarroa, Spain; Instituto de Agrobiotecnología (IdAB), CSIC-Gobierno de Navarra, Iruñako etorbidea 123, 31192 Mutiloabeti, Nafarroa, Spain; Instituto de Agrobiotecnología (IdAB), CSIC-Gobierno de Navarra, Iruñako etorbidea 123, 31192 Mutiloabeti, Nafarroa, Spain; Instituto de Agrobiotecnología (IdAB), CSIC-Gobierno de Navarra, Iruñako etorbidea 123, 31192 Mutiloabeti, Nafarroa, Spain; Instituto de Agrobiotecnología (IdAB), CSIC-Gobierno de Navarra, Iruñako etorbidea 123, 31192 Mutiloabeti, Nafarroa, Spain; Laboratory of Biochemistry, Faculty of Agriculture, Niigata University, Niigata 950-2181, Japan; Instituto de Agrobiotecnología (IdAB), CSIC-Gobierno de Navarra, Iruñako etorbidea 123, 31192 Mutiloabeti, Nafarroa, Spain; Instituto de Agrobiotecnología (IdAB), CSIC-Gobierno de Navarra, Iruñako etorbidea 123, 31192 Mutiloabeti, Nafarroa, Spain; Instituto de Agrobiotecnología (IdAB), CSIC-Gobierno de Navarra, Iruñako etorbidea 123, 31192 Mutiloabeti, Nafarroa, Spain; Centre of Region Haná for Biotechnological and Agricultural Research, Czech Advanced Technology and Research Institute, Olomouc, Czech Republic; Department of Chemical Biology, Faculty of Science, Palacký University, Olomouc CZ-78371, Czech Republic; Laboratory of Growth Regulators, Faculty of Science of Palacký University and Institute of Experimental Botany of the Czech Academy of Sciences, Olomouc CZ-78371, Czech Republic; Laboratory of Growth Regulators, Faculty of Science of Palacký University and Institute of Experimental Botany of the Czech Academy of Sciences, Olomouc CZ-78371, Czech Republic; Institute for Mediterranean and Subtropical Horticulture “La Mayora” (IHSM), CSIC-UMA, 29010 Málaga, Spain; Institute for Mediterranean and Subtropical Horticulture “La Mayora” (IHSM), CSIC-UMA, 29010 Málaga, Spain; Institute for Mediterranean and Subtropical Horticulture “La Mayora” (IHSM), CSIC-UMA, 29010 Málaga, Spain; Instituto de Agrobiotecnología (IdAB), CSIC-Gobierno de Navarra, Iruñako etorbidea 123, 31192 Mutiloabeti, Nafarroa, Spain; Institute for Mediterranean and Subtropical Horticulture “La Mayora” (IHSM), CSIC-UMA, 29010 Málaga, Spain

## Abstract

In Arabidopsis (*Arabidopsis thaliana*), the plastidial isoform of phosphoglucose isomerase (PGI1) mediates photosynthesis, metabolism, and development, probably due to its involvement in the synthesis of isoprenoid-derived signals in vascular tissues. Microbial volatile compounds (VCs) with molecular masses of <45 Da promote photosynthesis, growth, and starch overaccumulation in leaves through PGI1-independent mechanisms. Exposure to these compounds in leaves enhances the levels of *GLUCOSE-6-PHOSPHATE/PHOSPHATE TRANSLOCATOR2* (*GPT2*) transcripts. We hypothesized that the PGI1-independent response to microbial volatile emissions involves GPT2 action. To test this hypothesis, we characterized the responses of wild-type (WT), *GPT2*-null *gpt2-1*, *PGI1*-null *pgi1-2*, and *pgi1-2gpt2-1* plants to small fungal VCs. In addition, we characterized the responses of *pgi1-2gpt2-1* plants expressing *GPT2* under the control of a vascular tissue- and root tip-specific promoter to small fungal VCs. Fungal VCs promoted increases in growth, starch content, and photosynthesis in WT and *gpt2-1* plants. These changes were substantially weaker in VC-exposed *pgi1-2gpt2-1* plants but reverted to WT levels with vascular and root tip-specific *GPT2* expression. Proteomic analyses did not detect enhanced levels of GPT2 protein in VC-exposed leaves and showed that knocking out *GPT2* reduced the expression of photosynthesis-related proteins in *pgi1-2* plants. Histochemical analyses of GUS activity in plants expressing *GPT2-GUS* under the control of the *GPT2* promoter showed that *GPT2* is mainly expressed in root tips and vascular tissues around hydathodes. Overall, the data indicated that the PGI1-independent response to microbial VCs involves resetting of the photosynthesis-related proteome in leaves through long-distance GPT2 action.

## Teaser

The phosphoglucose isomerase1-independent response to microbial volatile compounds involves photosynthesis-related proteome resetting through long-distance glucose-6-P/phosphate translocator2 action.

## Introduction

Phosphoglucose isomerase (PGI) catalyzes the reversible isomerization of glucose-6-P (G6P) and fructose-6-P. This enzyme participates in the early steps of glycolysis and in the regeneration of G6P pools in the pentose phosphate pathway (PPP). In mammals, in addition to its role as a glycolytic and PPP enzyme, PGI plays moonlighting roles as a cytokine and growth factor (Chaput et al., 1988; Watanabe et al., 1996; Jeffery et al., 2000). Arabidopsis (*Arabidopsis thaliana*) has one PGI isozyme in the plastid, that is PGI1, which plays a key role in transitory starch production in mesophyll cells of leaves, connecting the Calvin–Benson cycle with the canonical starch biosynthetic pathway (Yu et al., 2000; Fünfgeld et al., 2022). Its activity is modulated by glycolytic and PPP metabolic intermediates (Dietz, 1985; Backhausen et al., 1997) and by its redox status (Heuer et al., 1982). PGI1 interacts with some plastid-localized members of the 14-3-3 family of proteins (McWhite et al., 2020; https://thebiogrid.org/13853/summary/arabidopsis-thaliana/pgi1.html), which regulate multiple biological processes by phosphorylation-dependent protein–protein interactions (Denison et al., 2011). Some phosphorylation sites of PGI1 are flanked by redox-sensitive cysteine residues that respond to environmental changes (Reiland et al., 2009; Wang et al., 2013; Liu et al., 2014; Yin et al., 2017; https://phosphat.uni-hohenheim.de/). It thus appears that PGI1 is subject to complex regulatory mechanisms.

PGI1-lacking *pgi1-2* plants display reduced photosynthetic capacity and slow growth phenotypes, and accumulate low levels of starch and fatty acids in leaves and seeds, respectively (Bahaji et al., 2015, 2018). Moreover, these plants accumulate low levels of isoprenoid hormones derived from the plastid-localized 2-C-methyl-D-erythritol 4-P (MEP) pathway that are important for growth, development, and photosynthesis including active forms of gibberellins and trans*-*zeatin (tZ)-type cytokinins (CKs; Bahaji et al., 2015, 2018). *PGI1* is mainly expressed in root tips and vascular tissues of cotyledons, mature leaves, and roots (Bahaji et al., 2018), where genes involved in the synthesis of MEP pathway-derived isoprenoid hormones are strongly expressed (Silverstone et al., 1997; Miyawaki et al., 2004; Mitchum et al., 2006; Behnam et al., 2013; Yang et al., 2020). Thus, we have proposed that PGI1 is an important determinant of photosynthesis, metabolism, growth, reproductive development, and seed yield, probably due to its involvement in the synthesis of storage reserves in the embryo and PPP/glycolytic metabolic intermediates necessary for the synthesis of MEP pathway-derived isoprenoid hormones in vascular tissues (Bahaji et al., 2015, 2018).

Microorganisms emit a plethora of volatile compounds (VCs) that promote plant growth and photosynthesis as well as strong developmental and metabolic changes (Zhang et al., 2008; Sánchez-López et al., 2016b; Martínez-Medina et al., 2017; Camarena-Pozos et al., 2019; Baroja-Fernández et al., 2021; Sharifi et al., 2022; Vlot and Rosenkranz, 2022). Recently, using a “box-in-box” in vitro co-cultivation system in which plants were grown in the vicinity of microbial cultures covered with charcoal filters, we showed that VCs with a molecular mass less than ca. 45 Da (hereinafter designated as “small VCs”) are important determinants of plant responses to microbial volatile emissions (Ameztoy et al., 2019, 2021; García-Gómez et al., 2019, 2020; Gámez-Arcas et al., 2022). Regulation of these responses is primarily nontranscriptional and involves global changes in the proteome (Ameztoy et al., 2021) and thiol redox proteome, particularly in photosynthesis- and starch biosynthesis-related proteins (Li et al., 2011; Ameztoy et al., 2019). Responses to small VCs also involve CK-mediated mechanisms wherein long-distance communication between roots and the aerial part of the plant play important roles (García-Gómez et al., 2019, 2020; Gámez-Arcas et al., 2022). Like in wild-type (WT) plants, small VCs promote growth, photosynthesis, and tZ accumulation in *pgi1-2* plants (Sánchez-López et al., 2016a). These compounds also promote the accumulation of exceptionally high levels of starch in *pgi1-2* leaves (Sánchez-López et al., 2016a). Therefore, the response of plants to small VCs involves PGI1-independent mechanisms, including the activation of an as-yet unidentified noncanonical starch biosynthetic pathway(s) in mesophyll cells of leaves (Bahaji et al., 2011; Baroja-Fernández et al., 2012; Sánchez-López et al., 2016a).

A striking alteration in the transcriptome of leaves of small fungal VC-treated plants involves strong up-regulation of levels of transcripts of *GPT2* (At1g61800; Sánchez-López et al., 2016b), a gene that codes for a plastidial G6P/Pi transporter (Kammerer et al., 1998). *GPT2* is implicated in dynamic photosynthetic acclimation to environmental changes, such as increased irradiance through mechanisms involving signaling of G6P partitioning between chloroplasts and the cytosol, and resetting of the photosynthesis-related proteome (Athanasiou et al., 2010; Dyson et al., 2015; Miller et al., 2017; Karim, 2021). Dyson et al. (2014) have suggested that *GPT2* plays an important role in sugar sensing or signaling during germination and the transition from heterotrophic to autotrophic growth in developing seedlings. At the transcript level, *GPT2* has low, almost undetectable expression in WT leaves (Athanasiou et al., 2010; Weise et al., 2019; https://bar.utoronto.ca/eplant), but is induced in starch-deficient mutants (Kunz et al., 2010). In leaves, different abiotic stress treatments promote the accumulation of *GPT2* transcripts in vascular and epidermal cells, but not in the mesophyll (Berkowitz et al., 2021). Elevated photosynthesis, phosphate starvation, or exogenous sugar supply upregulate *GPT2* transcript levels (Hammond et al., 2003; Gonzali et al., 2006; Athanasiou et al., 2010; van Dingenen et al., 2016; García-Gómez et al., 2019; Weise et al., 2019) and promote starch accumulation (Makino et al., 1999; Athanasiou et al., 2010; Lei et al., 2011). In addition, *35S* promoter-driven *GPT2* expression restores to the WT the low starch content phenotype of *pgi1-2* leaves (Niewiadomski et al., 2005). It is thus conceivable that the accumulation of high levels of starch in leaves of WT and *pgi1-2* plants promoted by small microbial VCs is due, at least partly, to enhanced GPT2-mediated incorporation of cytosolic G6P into the chloroplasts and subsequent conversion into starch, thus bypassing the PGI1 reaction (Sánchez-López et al., 2016b). Furthermore, because PGI1 is strongly expressed in vascular tissues and root tip cells (Bahaji et al., 2018), it is likely that changes promoted by small VCs in leaves are due to enhanced GPT2-mediated incorporation of cytosolic G6P into nonphotosynthetic plastids of vascular tissues and root tip cells and subsequent PGI1-mediated metabolization into growth and photosynthesis determinants including isoprenoid hormones. To test these hypotheses and clarify the mechanisms involved in plant responses to small microbial VCs, we compared the growth, photosynthetic, starch, and tZ contents as well as proteomic responses of WT, *GPT2*-null *gpt2-1*, *PGI1*-null *pgi1-2*, and *pgi1-2gpt2-1* plants to small VCs emitted by the fungal phytopathogen *Alternaria alternata.* We also characterized the response of *pgi1-2gpt2-1* plants ectopically expressing *GPT2* under the control of the vascular tissue-specific *Athspr* promoter (Zhang et al., 2014) to small VCs. Moreover, using plants transformed with constructs carrying the *GPT2* promoter fused to the *GUS* reporter, we examined the *GPT2* expression pattern. Results presented in this work provide strong evidence that, under conditions in which PGI1 activity is reduced, long-distance action of GPT2 plays an important role in the response of plants to small VCs through mechanisms involving resetting of the photosynthesis-related proteome in leaves. Evidence is provided that *GPT2* is subject to complex regulatory mechanisms that impede its expression in mesophyll cells of leaves.

## Results

### The response of *pgi1-2gpt2-1* plants to small fungal VCs is weaker than that of WT and *pgi1-2* plants

**Figure 1:**
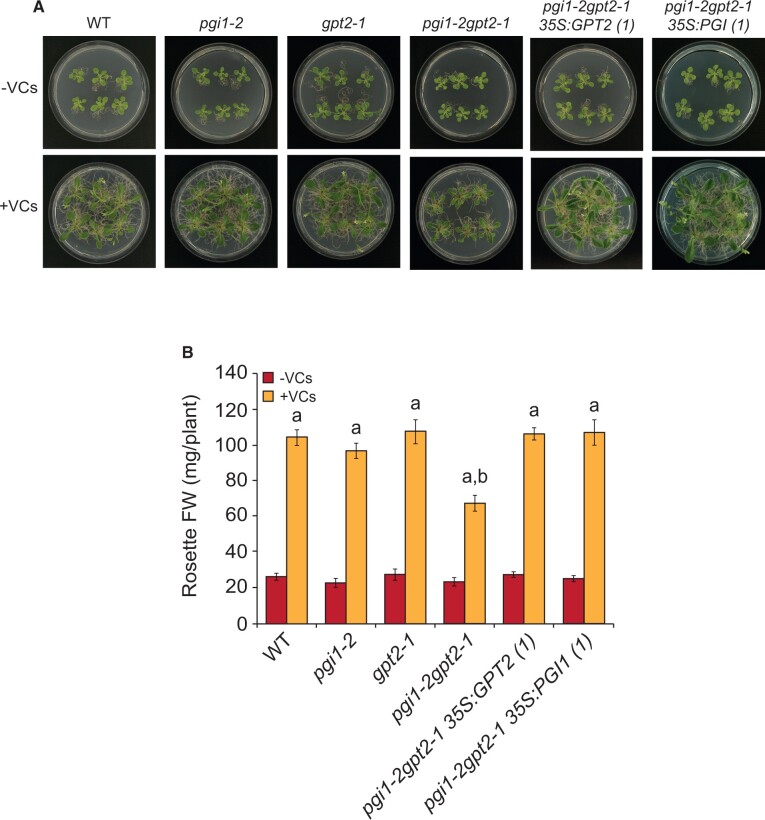
The growth response of *pgi1-2gpt2-1* plants to small fungal VCs is weaker than that of WT and *pgi1-2* plants. A, External phenotypes and (B) rosette FW of WT, *pgi1-2, gpt2-1*, and *pgi1-2gpt2-1* plants, and plants from one representative line each of *pgi1-2gpt2-1* transformed with *35S:GPT2* or *35S:PGI1* (*pgi1-2gpt2-1 35S:GPT2(1)* and *pgi1-2gpt2-1 35S:PGI1(1)*, respectively) cultured in the absence or continuous presence of small fungal VCs for 1 week. Values of rosette fresh weight (FW) in (B) are means ± se for three biological replicates (each a pool of 12 plants) obtained from four independent experiments. Lowercase letters indicate significant differences, according to Student’s *t* test (*P* < 0.05) between: “a” VC-treated and nontreated plants, and “b” VC-treated WT and mutant plants.

We compared growth, starch accumulation and photosynthesis responses of WT, *gpt2-1*, *pgi1-2*, and *pgi1-2gpt2-1* plants (Table 1) to VCs of molecular masses of less than ca. 45 Da emitted by adjacent *A. alternata* cultures. As shown in Figure 1, in the absence of small fungal VCs, the sizes and weights of rosettes of these plants were comparable to each other. Small fungal VCs strongly promoted rosette growth in WT, *pgi1-2* and, to a lesser extent, *pgi1-2gpt2-1* plants (Figure 1). The relatively weak promotion of growth of *pgi1-2gpt2-1* plants by small fungal VCs could be rescued by the ectopic expression of *PGI1* or *GPT2* under the control of the *35S* promoter (Figure 1).

**Table 1:** Plants used in this work

Designation	Description	Source
Wasilewskija-2 (Ws-2)	Wild-type	N1601
*pgi1-2*	*PGI1* knockout mutant	Kunz et al. (2010)
*gpt2-1*	*GPT2* knockout mutant	Niewiadomski et al. (2005)
*pgi1-2gpt2-1*	*pgi1-2* and *gpt2-1* double mutant	Bahaji et al. (2015)
*pgi1-2gpt2-1 35S:PGI1*	*pgi1-2gpt2-1* mutant expressing *PGI1* under the control of the cauliflower mosaic virus 35S promoter	This work
*pgi1-2gpt2-1 35S:GPT2*	*pgi1-2gpt2-1* mutant expressing *GPT2* under the control of the cauliflower mosaic virus 35S promoter	This work
*pgi1-2gpt2-1 promAthspr:GPT2*	*pgi1-2gpt2-1* mutant expressing *PGI1* under the control of the vascular tissue- and root tip-specific *Athspr* promoter	This work
*promAthspr:GUS*	WT plants expressing *GUS* under the control of the vascular tissue- and root tip-specific *Athspr* promoter	This work
*promGPT2:GUS*	WT plants expressing *GUS* under the control of the *GPT2* promoter	This work
*promGPT2:GPT2-GUS*	WT plants expressing translationally fused GPT2-GUS under the control of the *GPT2* promoter	This work
*35S:GPT2-GUS*	WT plants expressing translationally fused GPT2-GUS under the control of the cauliflower mosaic virus 35S promoter	This work

**Figure 2:**
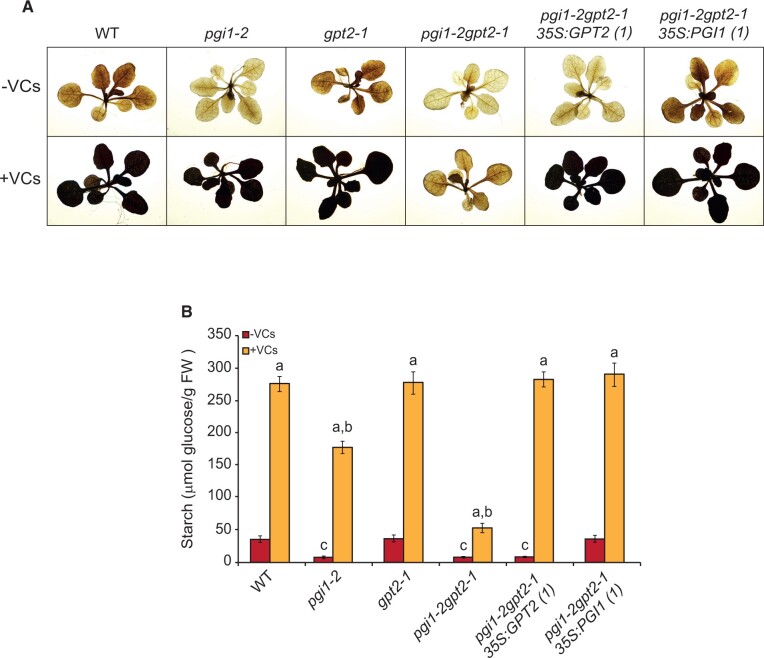
The starch accumulation response of *pgi1-2gpt2-1* plants to small fungal VCs is weaker than that of WT and *pgi1-2* plants. A, Iodine staining and (B) starch content in leaves of WT, *pgi1-2, gpt2-1*, and *pgi1-2gpt2-1* plants and plants from one representative line each of *pgi1-2gpt2-1* transformed with *35S:GPT2* or *35S:PGI1* (*pgi1-2gpt2-1 35S:GPT2(1)* and *pgi1-2gpt2-1 35S:PGI1(1)*, respectively) cultured in the absence or continuous presence of small VCs for 1 week. Values in (B) are means ± se for three biological replicates (each a pool of 12 plants) obtained from four independent experiments. Lowercase letters indicate significant differences, according to Student’s *t* test (*P* < 0.05) between: “a” VC-treated and nontreated plants, “b” VC-treated WT plants and mutants, and “c” VC nontreated WT and mutant plants.

In the absence of small fungal VCs, the starch content in mature leaves of *gpt2-1* plants was comparable to that of WT plants, as revealed by starch iodine staining (Figure 2A) and quantitative starch content measurement (Figure 2B) analyses. In keeping with Bahaji et al. (2015), the starch content in *pgi1-2* and *pgi1-2gpt2-1* mature leaves was ∼15% of that of WT leaves (Figure 2). The “low starch content” phenotype of *pgi1-2gpt2-1* plants could be rescued by the ectopic expression of *PGI1* under the control of the *35S* promoter but not by that of *GPT2*. Small fungal VCs promoted the accumulation of exceptionally high levels of starch in leaves of exposed WT and *gpt2-1* plants (Figure 2). In keeping with Sánchez-López et al. (2016b), these compounds also induced strong accumulation of starch in leaves of *pgi1-2* plants, although to a lesser extent than in leaves of WT plants (Figure 2). Small VCs increased the starch content in leaves of *pgi1-2gpt2-1* plants to levels much lower than those of VC-exposed *pgi1-2* leaves and comparable to those of WT leaves not exposed to small VCs (Figure 2). The weak promotion of starch accumulation by small VCs in leaves of *pgi1-2gpt2-1* plants could be rescued to WT levels by the ectopic expression of either *PGI1* or *GPT2* under the control of the *35S* promoter (Figure 2).

**Figure 3:**
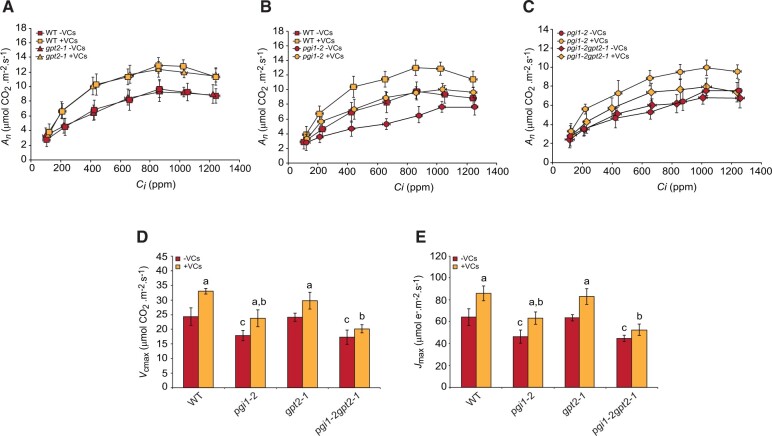
The photosynthetic response of *pgi1-2gpt2-1* plants to small fungal VCs is weaker than that of WT and *pgi1-2* plants. Curves of net CO_2_ assimilation rate (*A_n_*) versus intercellular CO_2_ concentration (*C_i_*) in leaves of (A) WT and *gpt2-1* plants, (B) WT and *pgi1-2* plants, and (C) *pgi1-2* and *pgi1-2gpt2-1* plants cultured in the absence or continuous presence of small VCs released by adjacent *A. alternata* cultures for 3 days. D, *V*_cmax_ and (E) *J*_max_ values calculated from the *A_n_/C_i_* curves. Treatment started 28 days after sowing plants. In (A–C), values are means ± se for four plants. In (D) and (E), values are means ± se for four biological replicates (each a pool of four plants) obtained from four independent experiments. Lowercase letters indicate significant differences, according to Student’s *t* test (*P* < 0.05), between: “a” VC-treated and nontreated plants, “b” VC-treated WT and mutants, and “c” VC nontreated WT and mutant plants.

In the absence of small VCs, values of the net rates of CO_2_ assimilation (*A_n_*) at all intracellular CO_2_ concentration (*C_i_*) levels, the maximum rate of carboxylation by Rubisco (*V*_cmax_) and the maximum electron transport demand for RuBP regeneration (*J*_max_) in *gpt2-1* plants were comparable to those of WT plants (Figure 3, A, D, and E). In *pgi1-2* plants, these values were lower than those in WT plants (Figure 3, B, D, and E), consistent with Bahaji et al. (2015), and similar to those of *pgi1-2gpt2-1* plants (Figure 3, C, D, and E). As expected, small VCs enhanced *A_n_* values at all *C_i_* levels as well as *V*_cmax_ and *J*_max_ values in WT plants (Figure 3, A, D, and E). Values of these photosynthetic parameters in small VC-treated *gpt2-1* plants were comparable to those of VC-treated WT plants (Figure 3, A, D, and E). In *pgi1-2* plants, small VCs enhanced values of *A_n_* at all *C_i_* levels as well as *V*_cmax_ and *J*_max_ to those of VC-non-treated WT plants (Figure 3, B, D, and E). Small fungal VCs induced a small, statistically nonsignificant increase of *A_n_*, *V*_cmax_, and *J*_max_ values in *pgi1-2gpt2-1* plants (Figure 3, C, D, and E). In both presence and absence of small fungal VCs, the “low photosynthetic capacity” phenotype of *pgi1-2gpt2-1* plants could be restored to almost WT levels by ectopic expression of *PGI1* or *GPT2* under the control of the *35S* promoter (Supplemental Figure S1).

### Knocking out *GPT2* decreases the content of tZ in *pgi1-2* plants

Having established *GPT2’*s involvement in the *pgi1-2* growth, photosynthetic, and starch accumulation responses to small fungal VCs, we compared the effects of these compounds on the tZ contents in *pgi1-2* and *pgi1-2gpt2-1* plants. For this, we measured the tZ contents in mature leaves of *pgi1-2* and *pgi1-2gpt2-1* plants cultured in the absence or continuous presence of small fungal VCs. We also measured the tZ contents in leaves of WT plants. Under both experimental conditions, the tZ content in *pgi1-2gpt2-1* leaves (0.71 ± 0.10 and 1.87 ± 0.18 pmol g^−1^ DW in plants cultured in the absence and presence of VCs, respectively) was substantially lower than in *pgi1-2* plants (1.59 ± 0.11 and 2.34 ± 0.27 pmol g^−1^ DW in plants cultured in the absence and presence of VCs, respectively), which in turn accumulated lower levels of tZ than WT leaves (2.54 ± 0.52 and 3.60 ± 0.05 pmol g^−1^ DW in plants cultured in the absence and presence of VCs, respectively).

### Vascular tissue- and root-tip-specific expression of *GPT2* is sufficient to revert to WT the poor response of *pgi1-2gpt2-1* plants to small VCs

**Figure 4:**
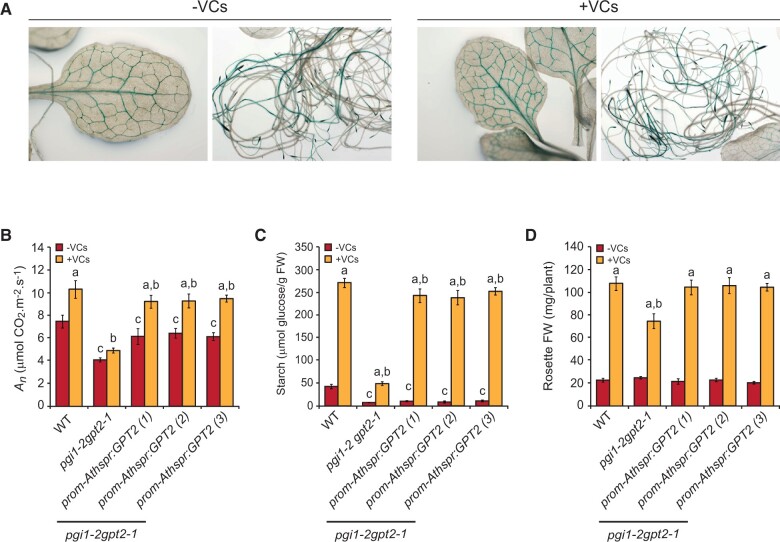
Vascular tissue- and root tip-specific expression of *GPT2* is sufficient to revert the poor photosynthetic, starch, and growth responses of *pgi1-2gpt2-1* plants to small VCs to WT levels. A, Expression pattern of the *Athspr* promoter in transgenic *promAthspr:GUS* plants cultured in the absence or presence of small fungal VCs for 1 week, as manifested by GUS histochemical staining of leaves and roots. B, Net CO_2_ assimilation rate (*A_n_*) at 400 ppm CO_2_, (C) starch content, and (D) rosette FW of WT, *pgi1-2gpt2-1*, and three independent lines of *pgi1-2gpt2-1* transformed with *promAthspr:GPT2* cultured in the absence or continuous presence of small VCs for 1 week. In (B), values are means ± se for four plants. Values in (C) and (D) are means ± se for three biological replicates (each a pool of 12 plants) obtained from four independent experiments. In (B–D), lowercase letters indicate significant differences, according to Student’s *t* test (*P* < 0.05), between: “a” VC-treated and nontreated plants, “b” VC-treated WT and mutants, and “c” VC nontreated WT and mutant plants.

*PGI1* is strongly expressed in root tips and vascular tissues of roots, cotyledons, hypocotyls, and fully expanded mature leaves (Bahaji et al., 2018). It is thus likely that vascular expression of *GPT2* plays an important role in the response of *pgi1-2* plants to small VCs. To test this hypothesis, we characterized *pgi1-2gpt2-1* plants transformed with *promAthspr:GPT2*, which express *GPT2* under the control of the vascular tissue-specific *Athspr* promoter (Zhang et al., 2014; Table 1). As shown in Figure 4A, preliminary histochemical analyses of *promAthspr:GUS* plants transformed with *promAthspr* fused to the *GUS* reporter showed vascular tissue and root tip specificity of *promAthspr*, both in the absence and presence of small VCs. Data obtained from three independent lines of *pgi1-2gpt2-1* plants transformed with *promAthspr:GPT2* revealed that, in the absence of small fungal VCs, vascular and root-tip-specific *GPT2* expression almost completely restored to WT levels the photosynthetic capacity of *pgi1-2gpt2-1* plants (Figure 4B) but did not restore the “low starch content” phenotype of these plants (Figure 4C). In the presence of small fungal VCs, vascular- and root tip-specific *GPT2* expression completely restored to WT levels the weight of VC-exposed *pgi1-2gpt2-1* plants (Figure 4D) and almost completely restored to WT levels the photosynthetic capacity and starch content of these plants (Figure 4, B and C).

### Knocking out *PGI1* and *GPT2* decreases the expression of photosynthesis-related proteins

**Figure 5:**
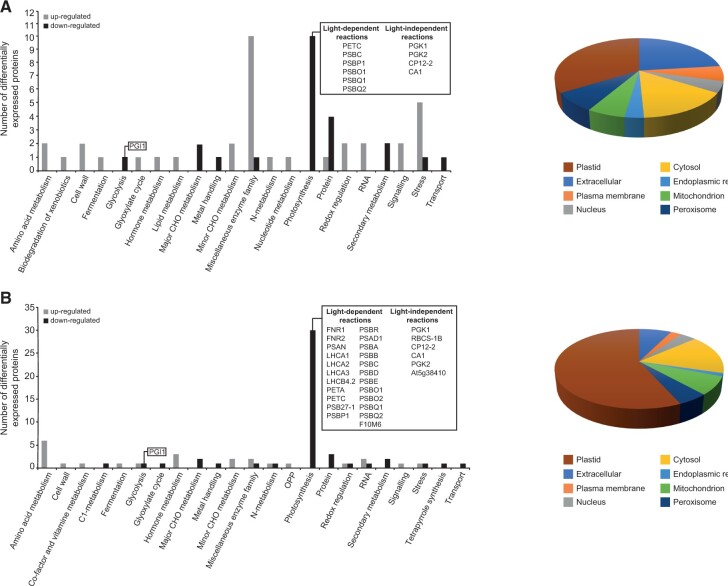
Knocking out *PGI1* and *GPT2* decreases the expression of photosynthesis-related proteins in small VC-exposed plants. The graphics represent the functional categorization of DEPs in the comparative study between leaves of (A) *pgi1-2* and WT plants and (B) *pgi1-2gpt2-1* and WT plants cultured in the presence of small VCs for two days. On the left side, proteins that were significantly down- or upregulated in mutants are arranged according to the putative functional category assigned by MapMan software. On the right side, DEPs are classified according to their subcellular localization. DEPs discussed here are shown in the boxes. The data were obtained from Supplemental Tables S6 and S8.

To obtain insights into the PGI1- and GPT2-mediated molecular mechanisms involved in the responses of plants to small VCs, we carried out high-throughput differential proteomic analyses between leaves of (1) WT plants cultured in the absence or presence of small VCs, (2) VC-exposed *gpt2-1* and VC-exposed WT plants, (3) VC-exposed *pgi1-2* and VC-exposed WT plants, and (4) VC-exposed *pgi1-2/gpt2-1* and VC-exposed WT plants. As a preliminary step to establish the VC exposure time for harvesting leaf samples, we carried out a time-course reverse transcription–quantitative polymerase chain reaction (RT–qPCR) analysis of *GPT2* transcript levels in leaves of WT plants cultured in the absence or presence of small VCs. We found that the pattern of *GPT2* transcript content in VC-exposed leaves was similar to that previously reported in leaves of plants exposed to increased irradiance (Athanasiou et al., 2010). During the first 16 h of VC exposure, *GPT2* transcript levels increased rapidly, and then fell to reach a steady-state substantially greater than that of the controls after 3 days of VC exposure (Supplemental Figure S2). Based on these observations, we decided to conduct proteomic analyses using leaves of plants exposed to small VCs for 2 days, which still exhibited high *GPT2* transcript levels. These analyses revealed that small fungal VCs promoted widespread proteome resetting in all genotypes analyzed. The results obtained can be summarized as follows:

- Four hundred twenty-five out of the 4,188 proteins identified in the comparative study between leaves of WT plants cultured in the absence or presence of small VCs were differentially expressed (Supplemental Tables S1 and S2). Using the broad characterizations outlined by the MapMan tool (https://mapman.gabipd.org/; Thimm et al., 2004), the proteins differentially expressed by small VCs were assembled into 29 functional groups (Supplemental Figure S3A). Predicted locations of these proteins using the SUBA4 Arabidopsis protein subcellular localization database (Hooper et al., 2017) included almost all cellular compartments, but the locations associated with the greatest number of proteins were the cytosol and plastid (Supplemental Table S1 and Supplemental Figure S3B). Nearly 70% of these proteins were identified as differentially expressed by small VCs in a previous differential proteomic study using a Col-O background (Ameztoy et al., 2021) (Supplemental Table S1). No statistically significant changes in the levels of GPT2 protein were observed upon small fungal VC treatment (Supplemental Table S2).
- Only 6 out of the 4,187 proteins identified in the comparative study between small VC-exposed *gpt2-1* leaves and VC-exposed WT leaves were differentially expressed (Supplemental Table S3). No statistically significant differences in GPT2 levels were observed between leaves of small VC-exposed *gpt2-1* and VC-exposed WT plants.
- Sixty-four out of the 4,186 proteins identified in the comparative study between VC-exposed *pgi1-2* leaves and VC-exposed WT leaves showed statistically different expression levels (Supplemental Tables S4 and S5; Figure 5A). Nearly 35% of these differentially expressed proteins (DEPs) were predicted to have a plastidial location and 10 of them were photosynthesis-related proteins (Supplemental Table S4; Figure 5A).
- Eighty-one out of the 4,148 proteins identified in the comparative study between VC-exposed *pgi1-2gpt2-1* leaves and VC-exposed WT leaves showed statistically significant different expression levels (Supplemental Tables S6 and S7; Figure 5B). Nearly 70% of these DEPs were predicted to have a plastidial location, and 29 of them were photosynthesis-related proteins (Supplemental Table S6; Figure 5B).

We next considered whether differences in the proteomes of VC-exposed *pgi1-2gpt2-1* leaves and VC-exposed WT leaves were due to differential perception and/or signaling of small VC or to knocking out of both *PGI1* and *GPT2*. We thus conducted differential proteomic analyses between leaves of *pgi1-2gpt2-1* and WT plants cultured in the absence of small fungal VCs. As shown in Supplemental Table S8 and Supplemental Figure S4, the majority of the proteins differentially expressed between leaves of *pgi1-2gpt2-1* and WT plants not exposed to VCs were also differentially expressed between leaves of small VC-exposed *pgi1-2gpt2-1* and WT plants (cf. Supplemental Table S6; Figure 5B). Therefore, we concluded that the reduced levels of photosynthesis-related proteins in VC-exposed *pgi1-2gpt2-1* plants were due to the lack of PGI1 and GPT2 rather than to differences in perception and/or signaling of small VC in the two genotypes.

### *GPT2* expression regulation

Proteomic data showing that small VCs did not enhance the GPT2 protein content in exposed leaves strongly indicated that *GPT2* expression is subjected to complex regulation. To test this hypothesis, we conducted RT–qPCR analyses of *GUS* transcript levels and GUS histochemical staining analyses in WT plants transformed with *promGPT2:GUS*, which expressed *GUS* under the control of the 1.1-kb *promGPT2* region immediately upstream the translation start codon of *GPT2* (Table 1). We also characterized plants transformed with *promGPT2:GPT2-GUS* and *35S:GPT2-GUS*, which expressed translationally fused GPT2-GUS under the control of *promGPT2* and the *35S* promoter, respectively (Table 1).

**Figure 6:**
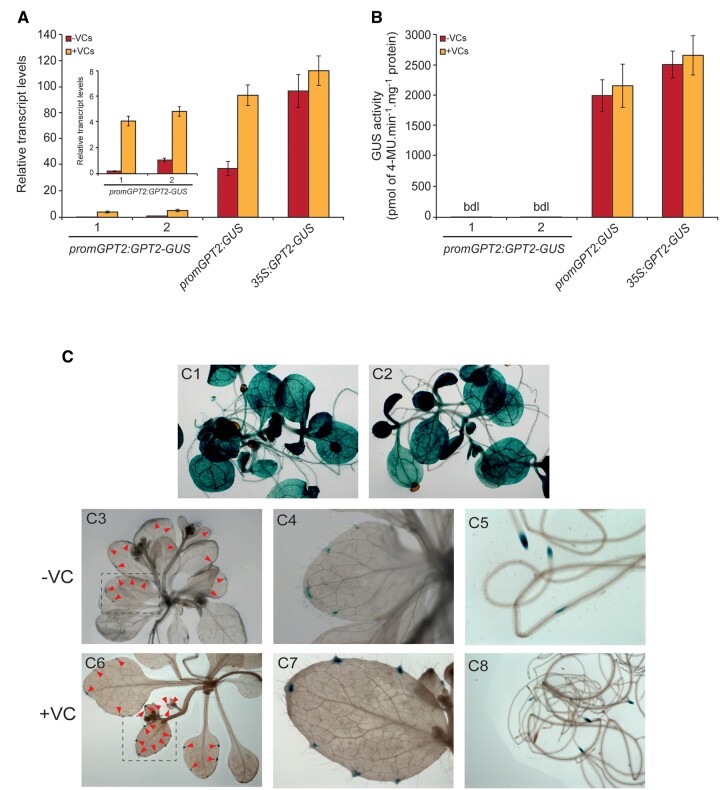
*GPT2* expression is subjected to complex regulation. A, Relative *GUS* transcript levels and (B) GUS activity in leaves of *promGPT2:GPT2-GUS, promGPT2:GUS*, and *35S:GPT2-GUS* plants cultured in the absence or presence of small VCs for 2 days. C, Histochemical localization of GUS activity in *promGPT2:GUS* (C1) and *35S:GPT2-GUS* (C2) plants cultured in the absence of small VCs, and *promGPT2:GPT2-GUS* plants cultured in the absence (C3–C5) or presence (C6–C8) of small VCs for two days. In “A”, the inset shows the relative *GUS* transcript levels in leaves of two independent representative lines of *promGPT2:GPT2-GUS* plants. Values in (A) and (B) are means ± se for three biological replicates (each a pool of 12 plants) obtained from four independent experiments. bdl: below detection limit, which was established at 2 pmol of 4-MU·min^−1^·mg^−1^ protein.

As shown in Figure 6A, *GUS* transcript levels in leaves of *promGPT2:GUS* plants not exposed to small VCs were approximately two-fold lower than in *35S:GPT2-GUS* leaves, indicating that the *promGPT2* sequence has strong promoter activity. However, *GUS* transcript levels in *promGPT2:GPT2-GUS* leaves were extremely lower than in *promGPT2:GUS* leaves, both in the absence and presence of small VCs. Exposure to small VCs enhanced *GUS* transcript levels in *promGPT2:GUS* and *promGPT2:GPT2-GUS* leaves, but not in *35S:GPT2-GUS* leaves (Figure 6A), indicating that *promGPT2* has the regulatory elements necessary for driving downstream gene expression in response to small VCs. Histochemical GUS activity analyses revealed that *promGPT2:GUS* and *35S:GPT2-GUS* plants exhibited strong GUS activity in all tissues and cell types of leaves and roots (Figure 6C). Regardless of the presence of small fungal VCs, different independent lines of *promGPT2:GPT2-GUS* plants showed detectable GUS activity mainly in root tips and vascular tissues around hydathodes, but not in other tissues such as the mesophyll of leaves (Figure 6C). Consistently, GUS activities in leaves of *promGPT2:GPT2-GUS* plants cultured in the absence or presence of VCs were negligible (Figure 6B). These results strongly indicated that *GPT2* expression is subject to complex regulatory mechanisms wherein *GPT2* coding sequences play important roles.

## Discussion

### *GPT2* is an important determinant of the response of *pgi1-2* plants but not of WT plants to small VCs

Plants adjust their photosynthetic processes to fluctuating environmental conditions to avoid photoinhibition and maximize yield through changes in the structure and composition of the photosynthetic apparatus (Gjindali et al., 2021). Such changes, referred to as dynamic photosynthetic acclimation, alter metabolism and endow plants with the necessary plasticity to withstand changes in their environment. Previous studies using *gpt2* plants have shown that exposure of leaves to increased irradiance enhances photosynthesis through a *GPT2*-mediated dynamic photosynthetic acclimation process, involving transient accumulation of *GPT2* transcripts and widespread reengineering of the leaf proteome (Athanasiou et al., 2010; Dyson et al., 2015; Miller et al., 2017). Here, we showed that enhancement of photosynthesis, growth and leaf starch content, and changes in the leaf proteome in *gpt2-1* plants promoted by small VCs are similar to those of WT plants (Figures 1–3; Supplemental Table S3). This strongly indicates that the molecular mechanisms involved in acclimation to increased irradiance and response to microbial VC exposure are different. We also showed that the response of *pgi1-2gpt2-1* plants to small VCs was weaker than that of *pgi1-2* plants (Figures 1–3). Moreover, the leaves of VC-exposed *pgi1-2gpt2-1* plants accumulated lower levels of a large number of photosynthesis-related proteins than VC-exposed *pgi1-2* leaves, which in turn accumulated lower levels of some of these proteins than VC-exposed WT leaves (Supplemental Tables S4–S7; Figure 5). The overall data indicate that (1) unlike in WT plants, *GPT2* plays an important role in the regulation of dynamic photosynthetic acclimation, growth, metabolism, and the expression of photosynthesis-related proteins in response to small fungal VCs in *pgi1-2* plants, and (2) the weak photosynthetic, growth and starch accumulation responses of *pgi1-2gpt2-1* plants to small VCs relative to WT and *pgi1-2* plants can be ascribed, at least partly, to reduce expression of photosynthesis-related proteins.

### The response of *pgi1-2* plants to small VCs involves *GPT2* but not enhanced levels of GPT2 protein in leaves

Small VCs promoted transitory accumulation of *GPT2* transcripts in leaves (Supplemental Figure S2), which may represent a case of activation of gene expression upon stress and subsequent decay during acclimation and restoration of homeostasis to a prestress state (Crisp et al., 2017; Garcia-Molina et al., 2020). Although transcript abundance on its own cannot be used to infer changes in the proteome and fluxes in central metabolism (Nakaminami et al., 2014; Schwender et al., 2014), this indicated that enhanced incorporation of cytosolic G6P into chloroplasts caused by increased GPT2 expression in leaves could be involved in the plant’s response to small VCs. However, our differential proteomic analyses did not detect any statistically significant accumulation of GPT2 protein in leaves promoted by small VCs (Supplemental Tables S1 and S2; Sánchez-López et al., 2016b; Ameztoy et al., 2021). These analyses also did not detect statistically significant higher levels of GPT2 protein in WT leaves than in *gpt2-1* and *pgi1-2gpt2-1* leaves (Supplemental Tables S3, S6, and S7). Moreover, histochemical GUS activity analyses of leaves of plants transformed with *promGPT2:GPT2-GUS* did not detect any enhancement of GUS activity promoted by small fungal VCs (Figure 6, B and C). Thus, the overall data indicated that (1) in keeping with the protein abundance database (https://pax-db.org/protein/612928), GPT2 protein levels in Arabidopsis leaves are marginally low and (2) in contrast to our initial hypothesis (Sánchez-López et al., 2016b), the response of *pgi1-2* plants to small VCs does not involve enhanced incorporation of cytosolic G6P into the chloroplast of leaf mesophyll cells caused by increased GPT2 expression.

### Vascular and root tip *GPT2* expression plays an important role in the PGI1-independent response to small VCs

**Figure 7:**
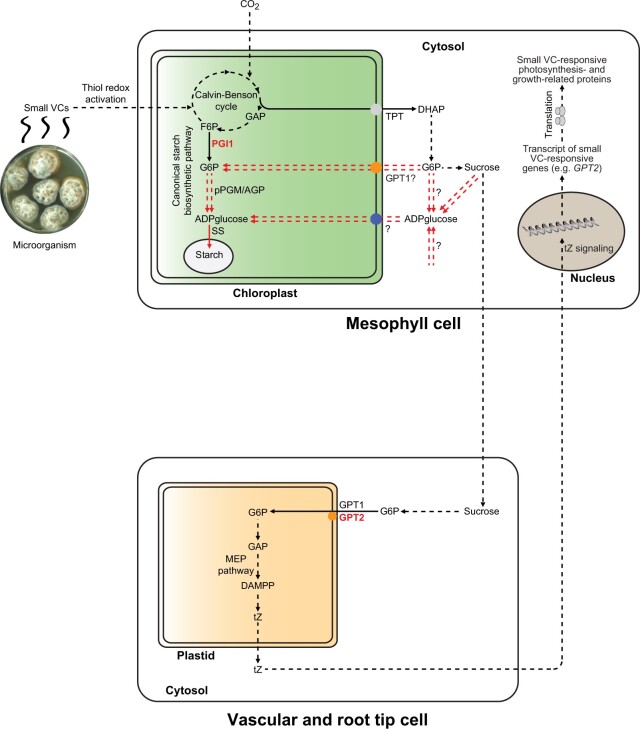
Suggested hypothetical model of regulation of the plant response to small fungal VCs by vascular and root tip GPT2 and PGI1 expression. According to this model, the response of plants to small fungal VCs involves mechanisms wherein signaling of both rapid thiol redox activation of photosynthesis in mesophyll cells of leaves (Ameztoy et al., 2019, 2021) and enhanced glycolytic or PPP activity in vascular tissues of roots play important roles. Thiol redox activation of photosynthesis promoted by small VCs increases the production of photosynthates (mainly sucrose), which are transported to vascular tissues and root tip cells and metabolized to G6P in the cytosol. This compound enters the plastid through the GPT transporters to fuel the plastid-localized glycolysis or PPP, where PGI1 participates in the metabolization of G6P. GAP produced by the PPP or glycolysis enters the MEP pathway to fuel the production of tZ, which is transported to mesophyll cells, where it initiates a cascade of reactions, leading to changes in the expression of photosynthesis-related proteins. This process guarantees a sustained high rate of photosynthesis and accelerated growth. According to this hypothetical model, VC-promoted starch overaccumulation in both WT and *pgi1-2* leaves could be a consequence of the stimulation of metabolic flux through noncanonical starch biosynthetic pathway(s) (highlighted in double dashed lines) that bypass PGI1 through the transport of cytosolic hexoses (e.g. G6P and/or ADPglucose) into the chloroplasts. pPGM: plastidial phosphoglucomutase; AGP: ADPglucose pyrophosphorylase; SS: starch synthase; TPT: triose-P transporter.

MEP pathway-derived tZ-type CKs are mainly synthesized in the root tips and the vascular tissues and then transported to shoots, where they regulate growth and processes including the expression of photosynthesis-related proteins and the photosynthetic acclimation to environmental changes (Miyawaki et al., 2004; Aloni et al., 2005; Boonman et al., 2007; Žd’árská et al., 2013; Kieber and Schaller, 2014; Ko et al., 2014; Cortleven and Schmülling, 2015). Root tips, vascular tissues, and hydathodes express *PGI1* and genes involved in rate-limiting steps of plastidic CK biosynthesis, translocation, and signaling (Bürkle et al., 2003; Miyawaki et al., 2004; Ferreira and Kieber, 2005; Bahaji et al., 2018). Here, we showed that tZ levels in VC-exposed *pgi1-2gpt2-1* leaves were lower than in VC-exposed *pgi1-2* leaves, which in turn accumulated lower levels of tZ than VC-exposed WT leaves. In addition, we found that *GPT2* is expressed in root tips and leaf vascular tissues around hydathodes, which are considered as transfer stations of CKs between xylem and phloem (Bürkle et al., 2003; Aloni et al., 2005; Nagawa et al., 2006). Furthermore, we found that vascular- and root tip-specific *GPT2* expression is sufficient to almost completely restore to WT levels the poor growth, photosynthetic, and starch accumulation responses of *pgi1-2gpt2-1* plants to small VCs (Figure 4). Therefore, the overall data indicated that the expression of PGI1 and GPT2 in root tips and vascular cells plays key roles in the response of plants to small VCs through mechanisms that harmonize the carbon status of the plant with growth, photosynthesis and metabolism. One such mechanisms could involve the provision of plastids of vascular and root tip cells with G6P derived from the metabolization of sucrose coming from leaves to fuel glycolysis or the PPP and provide precursors for the synthesis of MEP pathway-derived tZ, which once transported to leaves, initiate a cascade of signaling reactions, leading to changes in the expression of photosynthesis- and growth-related proteins (Figure 7). According to this view, *GPT2* expression could play an important role in the response of plants to small VCs under conditions in which G6P-metabolizing PGI1 activity is low. Yeasts, plants and animal cells possess transporter-like proteins, designated as transceptors, that act as receptors involved in nutrient sensing (Ho et al., 2009; Lima et al., 2010; Yang et al., 2012; Zhang et al., 2014; Volpe et al., 2016; Steyfkens et al., 2018). So far, no sugar transceptor has been identified in plants. We speculate that GPT2 could act as a G6P receptor for long-distance signaling of the carbon status of the plant under changing environmental conditions. However, further work is necessary to test this hypothesis.

Unlike in *pgi1-2gpt2-1* plants, small VCs promoted the accumulation of exceptionally high levels of starch in the mesophyll of *pgi1-2* leaves (Figure 2). This, and the fact that small VCs enhanced *GPT2* transcript levels, may in principle indicate that these compounds activate a non-canonical starch biosynthetic pathway(s) involving GPT2-mediated incorporation of cytosolic G6P, which once in the chloroplast of mesophyll cells of *pgi1-2* leaves is converted to starch. However, this idea conflicts with the facts that (1) leaves accumulate negligible levels of GPT2 protein both in the presence and absence of small VCs (Supplemental Tables S2 and S3; Figure 6C) and (2) vascular- and root-tip-specific *GPT2* expression strongly enhanced the starch content in leaves of small VC-exposed *pgi1-2gpt2-1* plants (Figure 4). It is thus conceivable that the accumulation of high levels of starch in leaves of small VC-exposed *pgi1-2* plants and *pgi1-2gpt2-1* plants specifically expressing *GPT2* in vascular tissues is due to both uptake of cytosolic hexoses into the chloroplast through non-GPT2 transporter system(s) and enhanced photosynthesis promoted by proteome resetting mechanisms, wherein vascular and root tip *GPT2* expression plays an important role (Figure 7). Regarding the mechanism(s) of uptake of cytosolic hexoses into the chloroplasts that can act as precursors for the synthesis of starch in leaves of small VC-exposed plants, it should be noted that plastids from Arabidopsis have two functional G6P/Pi translocators: GPT1 and GPT2 (Kammerer et al., 1998). GPT1 plays an important role in starch biosynthesis in floral tissues and guard cells (Hedhly et al., 2016; Flütsch et al., 2022). Histochemical analyses of GUS activity in plants expressing *GUS* under the control of the *GPT1* promoter showed GUS activity in mesophyll cells of leaves, indicating that *GPT1* is expressed in the mesophyll (Niewiadomski et al., 2005). However, like GPT2*, GPT1* transcript and protein levels are extremely low in mesophyll cells, as visualized using the Plant eFP browser (https://bar.utoronto.ca/eplant) and the PAXdb: Protein Abundance Database (https://pax-db.org/protein/633665) and GPT1 immunoblot analyses of leaves (cf. figure 7 in Baune et al., 2020). Furthermore, small microbial VCs did not enhance *GPT1* transcript levels and GPT1 protein content in leaves (Sánchez-López et al., 2016b) (Supplemental Tables S2 and S5). Further work is necessary to test the possible involvement of GPT1 in the accumulation of exceptionally high levels of starch in microbial VC-exposed WT and *pgi1-2* leaves. Chloroplasts also have a glucose transporter (pGlcT; Weber et al., 2000) and hexokinase (Giese et al., 2005), potentially enabling the incorporation of cytosolic glucose and subsequent conversion into G6P. However, GlcT is involved in the export to the cytosol of glucose derived from the starch breakdown during the night, but not in the import of cytosolic glucose to the chloroplasts during illumination (Weber et al., 2000; Cho et al., 2011). Chloroplasts also possess a yet to be identified transporter of the starch precursor molecule, the ADPglucose (Pozueta-Romero et al., 1991; Bahaji et al., 2014). Microbial volatiles promote the accumulation of ADPglucose and starch in leaves of plants lacking plastidial enzymes of the canonical starch biosynthetic pathway involved in the synthesis of this compound (Bahaji et al., 2011), which would indicate that small microbial VCs stimulate cytosolic ADPglucose production. One possible source of cytosolic ADPglucose in leaves is sucrose synthase (SUS; Baroja-Fernández et al., 2012). However, recent studies have shown that leaves of SUS-lacking plants accumulate WT levels of ADPglucose (Fünfgeld et al., 2022). It is thus likely that starch biosynthesis in leaves of small VC-exposed *pgi1-2* plants and *pgi1-2gpt2-1* plants specifically expressing *GPT2* in vascular tissues involves, at least partly, the production of cytosolic ADPglucose through SUS-independent mechanisms and subsequent transport of this hexose into the chloroplast (Figure 7). However, further work is necessary to test these hypotheses.

### *GPT2* expression is subjected to complex regulation

Results presented in this work provide strong evidence that *GPT2* expression is subject to complex regulatory mechanisms. In the absence of small VCs, *GUS* transcript levels in *promGPT2:GUS* leaves were relatively high and comparable to those of *35S:GPT2-GUS* leaves (Figure 6A). This was rather surprising, as leaves not exposed to small VCs accumulate negligible levels of *GPT2* transcripts (Supplemental Figure S2; Weise et al., 2019). Noteworthy, *GUS* transcript levels in *promGPT2:GPT2-GUS* leaves were extremely lower than in *promGPT2:GUS* leaves (Figure 6A). Overall, the data indicate that *GPT2* expression is subject to mechanisms mediated by cooperatively acting regulatory elements located at both sides of the translation start ATG codon of the *GPT2* gene and/or at both sides of the translation start AUG codon of *GPT2* transcripts that impede accumulation of high *GPT2* transcript levels in leaves. The fact that small VCs enhanced *GPT2* transcript levels in leaves (Supplemental Figure S2) and *GUS* transcript levels in *promGPT2:GPT2-GUS* leaves (Figure 6A) would indicate that such mechanisms are partially inhibited by small VCs. Unlike *35S:GPT2-GUS* and *promGPT2:GUS* leaves showing strong GUS activity in all tissues (Figure 6C), GUS activity in *promGPT2:GPT2-GUS* leaves not exposed to small VCs was detectable only in vascular tissues around hydathodes, but not in other tissues such as the mesophyll (Figure 6C), which is consistent with the negligible accumulation of *GPT2* transcripts and GPT2 protein in the whole leaf. Small VCs did not promote accumulation of GPT2 protein in WT leaves (Supplemental Table S2) or GUS activity (Figure 6B) in *promGPT2:GPT2-GUS* leaves despite promoting accumulation of *GPT2* and *GPT2-GUS* transcripts, respectively (Supplemental Figure S2; Figure 6A). Overall, the data indicate that elements located around the translation start AUG codon of *GPT2* transcripts cooperatively act to impede GPT2 translation in VC-exposed mesophyll cells.

Epigenetic factors of control of gene transcription, such as small RNAs and DNA methylation, are relevant modulators of plantś responses to the environment and their biotic interactions (Lämke and Bäurle, 2017; Alonso et al., 2019). On the other hand, mechanisms of posttranscriptional control of gene expression, such as N^6^-methylation of adenosine (m^6^A), are important in controlling the stability and translatability of mRNAs (Arribas-Hernández and Brodersen, 2020). These mechanisms are affected by environmental factors, and strongly determine growth, development, and stress adaptation (Arribas-Hernández and Brodersen, 2020). Unlike WT plants, *met1* and *mta* mutants deficient in CG maintenance DNA methylation and m^6^A transcript modulation, respectively, accumulate high levels of *GPT2* transcripts (Lister et al., 2008; Bodi et al., 2012). Therefore, it is highly conceivable that both regulation of *GPT2* expression and the *GPT2*-mediated PGI1-independent response of plants to small VCs involves mechanisms wherein regulation of genomic *GPT2* DNA methylation and/or m^6^A transcript modulation play important roles. However, further work is necessary to evaluate these hypotheses.

### Additional remarks: enhanced photosynthesis is not the sole important determinant of enhanced growth and starch accumulation promoted by small fungal VCs

CKs are major determinants of photosynthesis and growth (Cortleven and Valcke, 2012; Kieber and Schaller, 2014). *pgi1-2* and *pgi1-2gpt2-1* plants exposed to small VCs were bigger and accumulated more starch than WT plants not exposed to small VCs, despite having comparable photosynthetic capacities (Figures 1–3). In addition, VC-promoted relative tZ content increase in *pgi1-2gpt2-1* leaves (2.6-fold) was higher than in *pgi1-2* and WT leaves (1.7- and 1.4-fold, respectively). This indicates that (1) factors other than relative increase of tZ content are important for enhancement of photosynthesis by microbial VCs and (2) photosynthesis is not the sole important determinant of growth and metabolic changes promoted by small VCs. This agrees with current ideas arguing against photosynthesis being the main rate-controlling factor for plant growth (Körner, 2015). Starch biosynthesis is subjected to redox regulation (Hendriks et al., 2003), and small fungal VCs redox-activate starch biosynthetic enzymes (Li et al., 2011; Ameztoy et al., 2019; García-Gómez et al., 2019), which could partly explain why small VC-exposed *pgi1-2* leaves, and to a lesser extent *pgi1-2gpt2-1* leaves, accumulated more starch than leaves of WT plants not exposed to VCs (Figure 2). In addition, VC-exposed WT, *pgi1-2*, and *pgi1-2gpt2-1* plants accumulated more reactive oxygen species scavengers, enzymes of the MEP, shikimate, and cytosolic glycolytic pathways, proteins involved in the synthesis of photosynthetic pigments, ribosomal proteins, and chaperones than leaves of WT plants not exposed to small VCs (Supplemental Figure S3; Figure 5). This could explain, at least in part, the higher growth of VC-treated WT, *pgi1-2*, and *pgi1-2gpt2-1* plants relative to that of non-VC-treated WT plants.

## Materials and methods

### Plants, growth conditions, and sampling

The work was carried out using Arabidopsis (*Arabidopsis thaliana* L., Heynh) WT plants (ecotype Wasilewskija-2, Ws-2), *pgi1-2* knockout mutants (Kunz et al., 2010), *gpt2-1* knockout mutants (GABI_454H06), and *pgi1-2gpt2-1* plants (Bahaji et al., 2015; Table 1). We also used plants from three independent lines each of *pgi1-2gpt2-1* plants expressing *PGI1* or *GPT2* under the control of the cauliflower mosaic virus 35S promoter (*pgi1-2gpt2-1 35S:PGI1* and *pgi1-2gpt2-1 35S:GPT2*, respectively; Table 1). In addition, we used plants from 10 independent lines each of WT plants expressing GUS under the control of the vascular tissue-specific *Athspr* promoter, which comprises the 1.67-kb region immediately upstream the translation start site of *Athspr* (Zhang et al., 2014) (*promAthspr:GUS*) and *pgi1-2gpt2-1* plants expressing *GPT2* under the control of *promAthspr* (*pgi1-2gpt2-1 promAthspr:GPT2*; Table 1). Moreover, we used plants from 10 independent lines each of WT plants expressing *GUS* under the control of the 1.1-kb region immediately upstream the translation start codon of *GPT2* (*promGPT2:GUS*; Table 1). Furthermore, we used plants expressing *GPT2-GUS* under the control of *promGPT2* and the 35S promoter (*promGPT2:GPT2-GUS* and *35S:GPT2-GUS*, respectively; Table 1). The *35S:PGI1, 35S:GPT2, promAthspr:GUS, promAthspr:GPT2, promGPT2:GUS, promGPT2:GPT2-GUS*, and *35S:GPT2-GUS* plasmid constructs were produced using Gateway technology as illustrated in Supplemental Figure S5 and confirmed by sequencing. Primers used for PCR amplification of *PGI1* and *GPT2* cDNA, *GUS*, and the *Athspr* and *GPT2* promoters are listed in Supplemental Table S9. The plasmid constructs were transferred to *Agrobacterium tumefaciens* EHA105 cells by electroporation and utilized to transform *Arabidopsis* plants as described by Clough and Bent (1998).

Seeds were sown and plants cultured in Petri dishes containing half-strength agar solidified Murashige and Skoog (MS) medium in growth chambers providing “long day” 16-h light (90 µmol photons s^−1^ m^−2^), 22°C/8-h dark, 18°C cycles. *Alternaria alternata* was cultured in Petri dishes as described in Sánchez-López et al. (2016a). Effects of small fungal VCs on plants were investigated using the “plasticized PVC wrap and charcoal filter-based box-in-box” co-cultivation system described in Gámez-Arcas et al. (2022). VC treatment started at 14 days after sowing growth stage of plants. At the indicated incubation periods, leaves were harvested, immediately freeze-clamped and ground to a fine powder in liquid nitrogen with a pestle and mortar.

### RT–qPCR analyses

RNA was extracted and reverse-transcribed essentially as described in Ameztoy et al. (2021). RT–qPCR amplification of *GPT2* and *GUS* genes was performed using primers listed in Supplemental Table S10.

### Determination of gas exchange rates and photosynthetic parameters

Gas exchange rates were determined as described by Sánchez-López et al. (2016b) using a LI-COR 6400 gas exchange portable photosynthesis system (LI-COR, Lincoln, NE, USA). *A_n_* was calculated as described by von Caemmerer and Farquhar (1981). *V*_cmax_ and *J*_max_ values were calculated from *A_n_*/*C_i_* curves according to Long and Bernacchi (2003).

### *GUS* expression analysis

Expression of the *GUS* reporter gene was monitored using the histochemical staining and fluorometric assays described by Jefferson et al. (1987).

### Iodine staining

Iodine staining of leaves was carried out as described by Bahaji et al. (2015).

### Analytical procedures

Levels of tZ were determined according to Novák et al. (2008). The total photosynthetic pigments content was quantified according to Lichtenthaler (1987). Starch was measured with an amyloglucosidase-based test kit (Boehringer Mannheim).

### Proteomic analysis

High-throughput, isobaric labeling-based differential proteomic analyses were conducted essentially as described in Sánchez-López et al. (2016a), but the tryptic peptides were labeled using a TMT6plex Isobaric Mass Tagging kit (Thermo Fischer Scientific). Statistical significance was measured using *q*-values (FDR). The cut-off for identifying DEPs was established at FDR ≤0.05% and log2 ratios (+VC treatment versus −VC treatment) of >0.3 (for proteins whose expression was upregulated by fungal VCs) or less than −0.3 (for proteins whose expression was downregulated by VCs).

### Statistical analysis

Unless otherwise indicated, presented data are means (±se) obtained from three to four independent experiments, with three replicates for each experiment. The significance of differences between plants VCs was statistically evaluated with Student’s *t* test using SPSS software. Differences were considered significant if *P* < 0.05.

### Accession numbers

Sequence data from this article can be found in the GenBank/EMBL data libraries under accession numbers NC_003075 and NC_003070 (for *PGI1* and *GPT2*, respectively).

## Supplemental data

The following materials are available in the online version of this article.

**Supplemental Figure S1.** Net CO_2_ assimilation rate (*A_n_*) at 400 ppm CO_2_ of WT and *pgi1-2gpt2-1* plants and plants from one representative line each of *pgi1-2gpt2-1* transformed with *35S:PGI1* or *35S:GPT2* (*pgi1-2gpt2-1 35S:PGI1(1)* and *pgi1-2gpt2-1 35S:GPT2(1)*, respectively) cultured in the absence or continuous presence of small VCs emitted by adjacent *A. alternata* cultures for 72 h.

**Supplemental Figure S2.** Time-course of *GPT2* transcript levels in leaves of WT plants cultured in the absence or continuous presence of small VCs emitted by adjacent *A. alternata* cultures for 160 h.

**Supplemental Figure S3.** Small VCs promote changes in the leaf proteome of WT plants.

**Supplemental Figure S4.** Knocking out *GPT2* and *PGI1* decreased the expression of photosynthesis-related proteins in leaves of plants not exposed to small VCs.

**Supplemental Figure S5.** Stages in the construction of the *35S:PGI1, 35S:GPT2, 35S:GPT2-GUS, promGPT2:GPT2-GUS, promGPT2:GUS, promAthspr:GPT2*, and *promAthspr:GUS* plasmids.

**Supplemental Table S1.** List of proteins differentially expressed by small fungal VCs in leaves of WT plants with a confident statistical significance level (small fungal VC-treated versus nontreated plants).

**Supplemental Table S2.** List of proteins identified in the comparative proteomic study between leaves of WT plants cultured in the absence or presence of fungal VCs.

**Supplemental Table S3.** List of proteins identified in the comparative proteomic study between leaves of WT and *gpt2-1* plants cultured in the presence of small fungal VCs.

**Supplemental Table S4.** List of DEPs identified in the comparative proteomic study between leaves of WT and *pgi1-2* plants cultured in the presence of small fungal VCs with a confident statistical significance level (*pgi1-2* versus WT).

**Supplemental Table S5.** List of proteins identified in the comparative proteomic study between leaves of WT and *pgi1-2* plants cultured in the presence of small fungal VCs.

**Supplemental Table S6.** List of DEPs identified in the comparative proteomic study between leaves of WT and *pgi1-2gpt2-1* plants cultured in the presence of small fungal VCs with a “confident” statistical significance level (*pgi1-2gpt2-1* versus WT).

**Supplemental Table S7.** List of proteins identified in the comparative proteomic study between leaves of WT and *pgi1-2gpt2-1* plants cultured in the presence of small fungal VCs.

**Supplemental Table S8.** List of DEPs identified in the comparative proteomic study between leaves of WT and *pgi1-2gpt2-1* plants cultured in the absence of small fungal VCs with a “confident” statistical significance level (*pgi1-2gpt2-1* versus WT).

**Supplemental Table S9.** Primers for PCR amplification used in this study.

**Supplemental Table S10.** Primers for RT–qPCR used in this study.

## Supplementary Material

kiac433_Supplementary_Data

## Notes

These authors contributed equally (S.G.-A. and F.J.M.).

F.J.M. and J.P-.R. designed the experiments and analyzed the data; S.G.-A., F.J.M., A.R.-B., A.M.S.-L., M.B., E.B.-F., A.B., G.A., N.D.D., K.D., O.N., J.L.-L., A.G.C., and R.J.L.M. performed most of the experiments and discussed the data; J.P.-R. and F.J.M. supervised the experiments; S.G.-A. and J.P.-R. wrote the article with contributions from all the authors; All authors contributed in the discussions, and reviewed and approved the final manuscript prior to submission; J.P.-R. and F.J.M. conceived the project and research plans.

The author responsible for distribution of materials integral to the findings presented in this article in accordance with the policy described in the Instructions for Authors (https://academic.oup.com/plphys/pages/general-instructions) is: Javier Pozueta-Romero (javier.pozueta@csic.es).
