# Supplementary material for: Glucose-6-P/phosphate translocator2 mediates the phosphoglucose-isomerase1-independent response to microbial volatiles
Source: Plant Physiol. 2022 Sep 16;190(4):2137–54. doi: 10.1093/plphys/kiac433 (PMC9706466; doi:10.1093/plphys/kiac433)
Supplement: kiac433_Supplementary_Data [file kiac433_supplementary_data.zip › kiac433_Supplementary_Data/Supplemental Data.pdf]

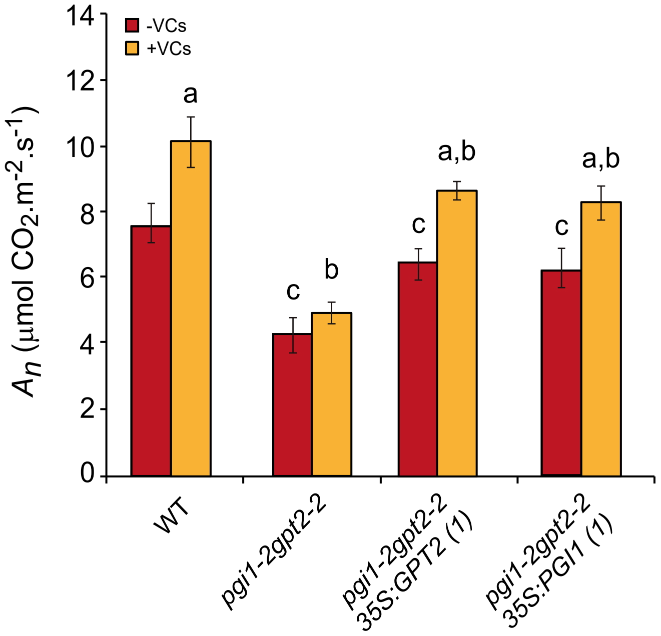

Supplemental Figure S1: Net CO_2 assimilation rate (A_n) at 400 ppm CO_2 of wild-type (WT) and *pgi1-2gpt2-1* plants and plants from one representative line each of *pgi1-2gpt2-1* transformed with *35S:PGI1* or *35S:GPT2* (*pgi1-2gpt2-1 35S:PGI1(1)* and *pgi1-2gpt2-1 35S:GPT2(1)*, respectively) cultured in the absence or continuous presence of small volatile compounds (VCs) emitted by adjacent *A. alternata* cultures for 72 hours. Values are means ± SE for three biological replicates (each a pool of 12 plants) obtained from four independent experiments. Lowercase letters indicate significant differences, according to Student´s t-test ($P<0.05$), between: "a" VC-treated and non-treated plants, "b" VC-treated WT and mutants, and "c" VC-non-treated WT plants and mutants.

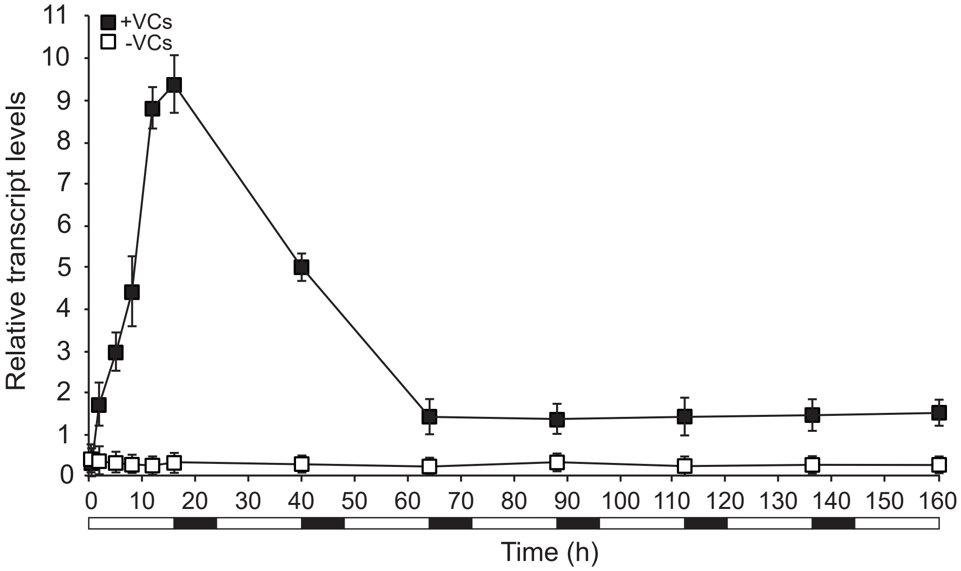

Supplemental Figure S2: Time-course of *GPT2* transcript levels in leaves of wild-type (WT) plants cultured in the absence or continuous presence of small volatile compounds (VCs) emitted by adjacent *A. alternata* cultures for 160 hours. Values are means ± SE for three independent experiments.

A

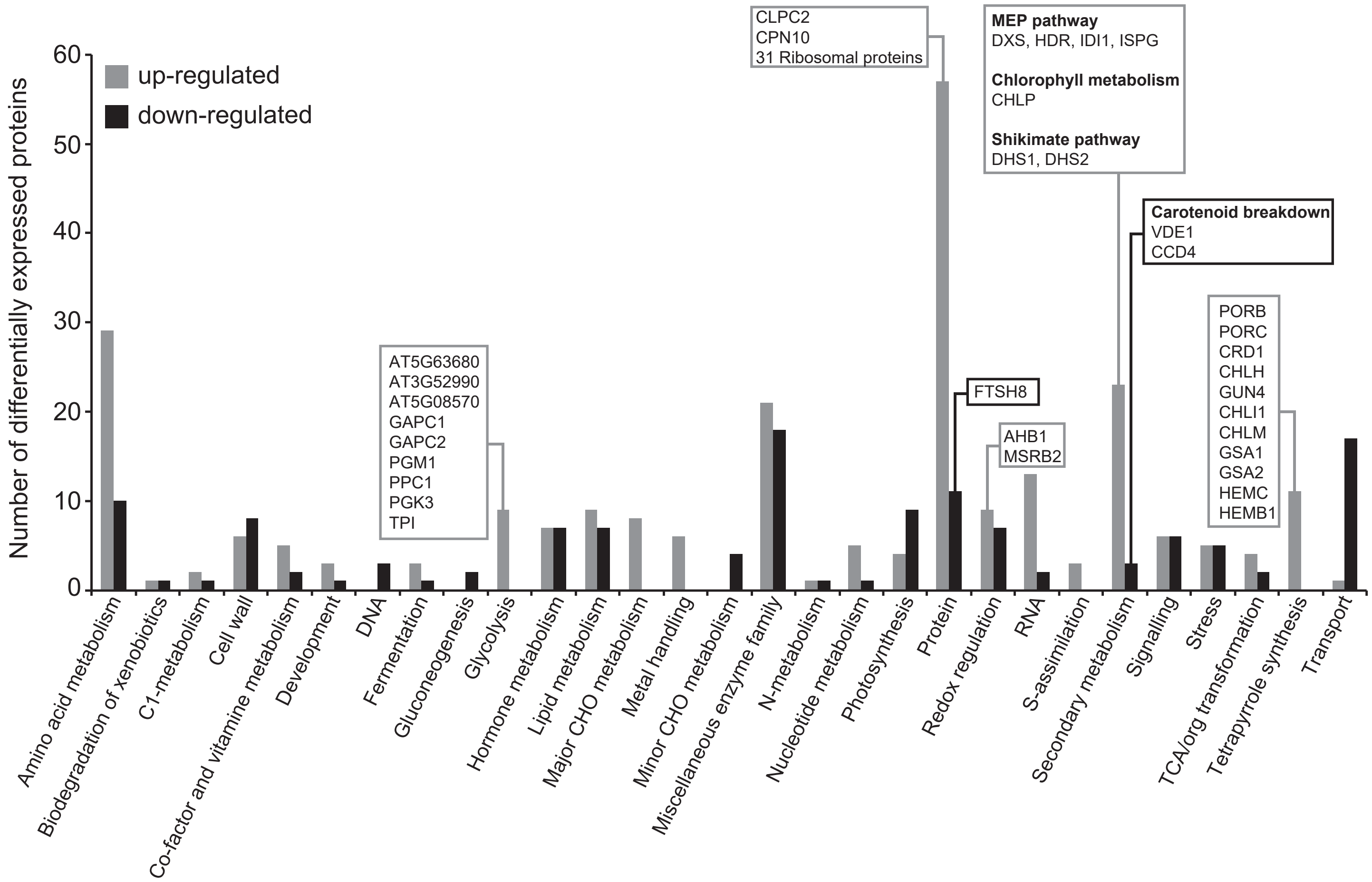

B

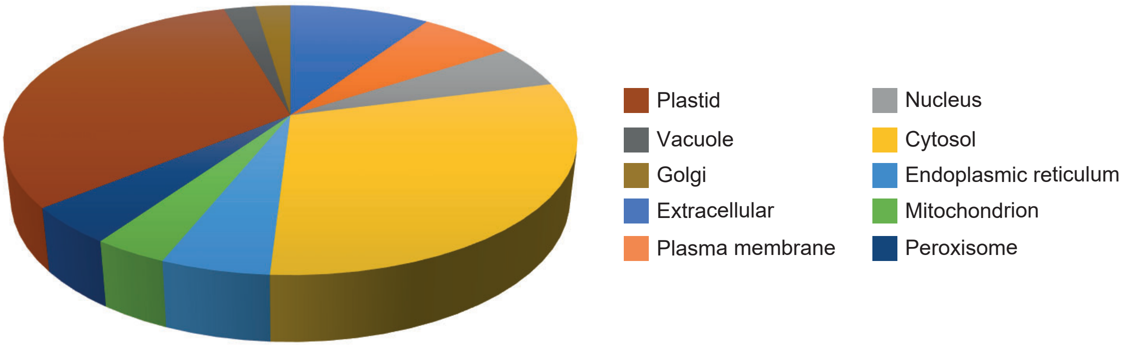

Supplemental Figure S3: Small volatile compounds (VCs) promote changes in the leaf proteome of wild-type (WT) plants. (A) Functional categorization of differentially expressed proteins (DEPs) in leaves of WT plants cultured in the presence of small VCs emitted by adjacent *A. alternata* cultures for 2 days. Proteins that were significantly down- or up-regulated following VC exposure are arranged according to the putative functional category assigned by MapMan software. The numbers of up- and down-regulated proteins in each categorical group are indicated by gray and black bars, respectively. DEPs discussed here are shown in the boxes. The data were obtained from **Supplemental Table S1**. (B) Classification of DEPs according to their predicted subcellular localization.

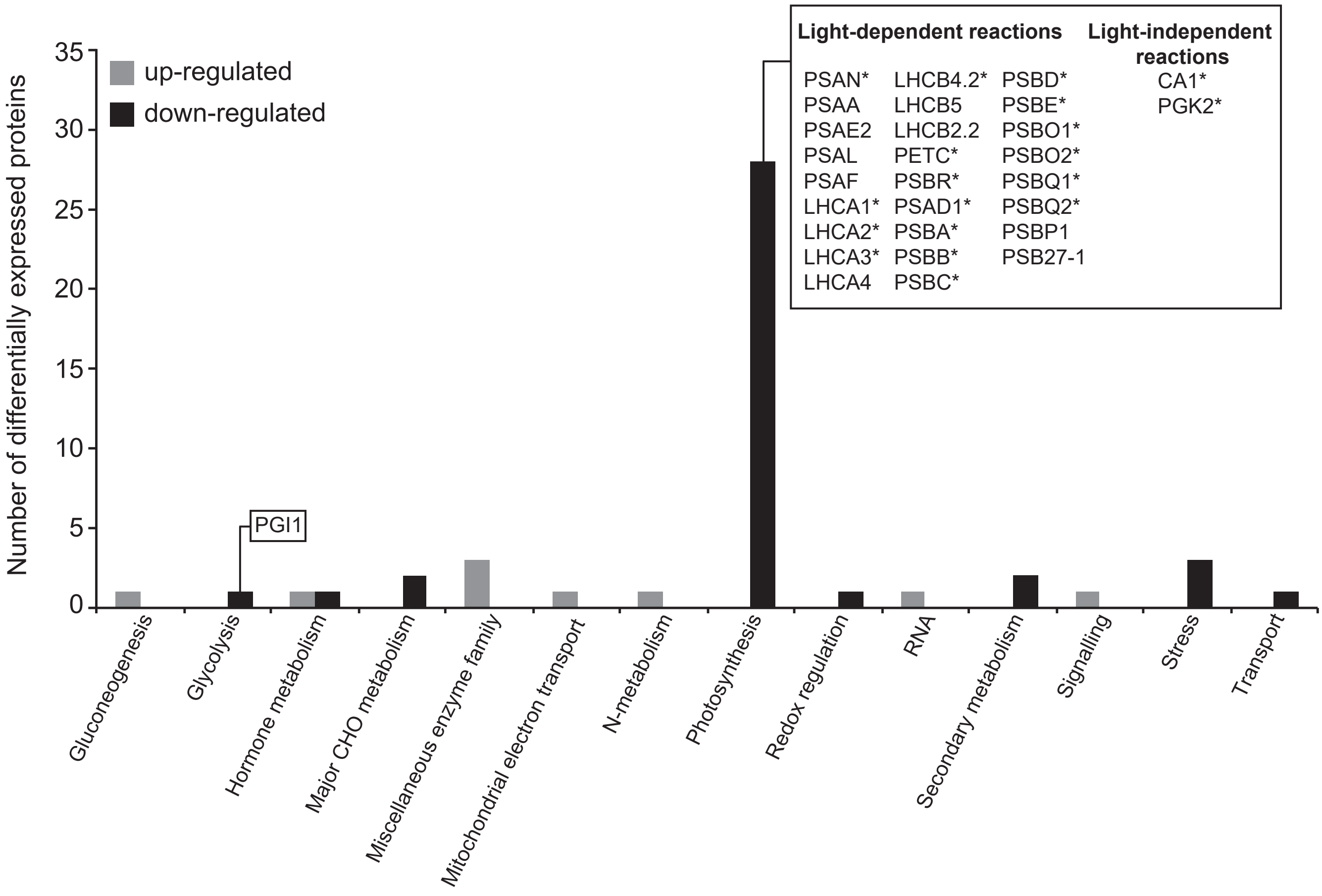

Supplemental Figure S4: Knocking out *GPT2* and *PGI1* decreased the expression of photosynthesis-related proteins in leaves of plants not exposed to small volatile compounds (VCs). The graphic represents the functional categorization of differentially expressed proteins (DEPs) in the comparative study between leaves of WT and *pgi1-2gpt2-1* plants cultured in the absence of small VCs emitted by adjacent *A. alternata* cultures for 2 days. Proteins that were significantly down- or up-regulated in *pgi1-2gpt2-1* plants are arranged according to the putative functional category assigned by MapMan software. The numbers of up- and down-regulated proteins in each categorical group are indicated by gray and black bars, respectively. The data were obtained from **Supplemental Table S8**. DEPs discussed here are shown in the boxes, and asterisks indicate DEPs identified in the comparative proteomic study between leaves of WT and *pgi1-2gpt2-1* plants cultured in the presence of small VCs (cf. **Figure 5B**).

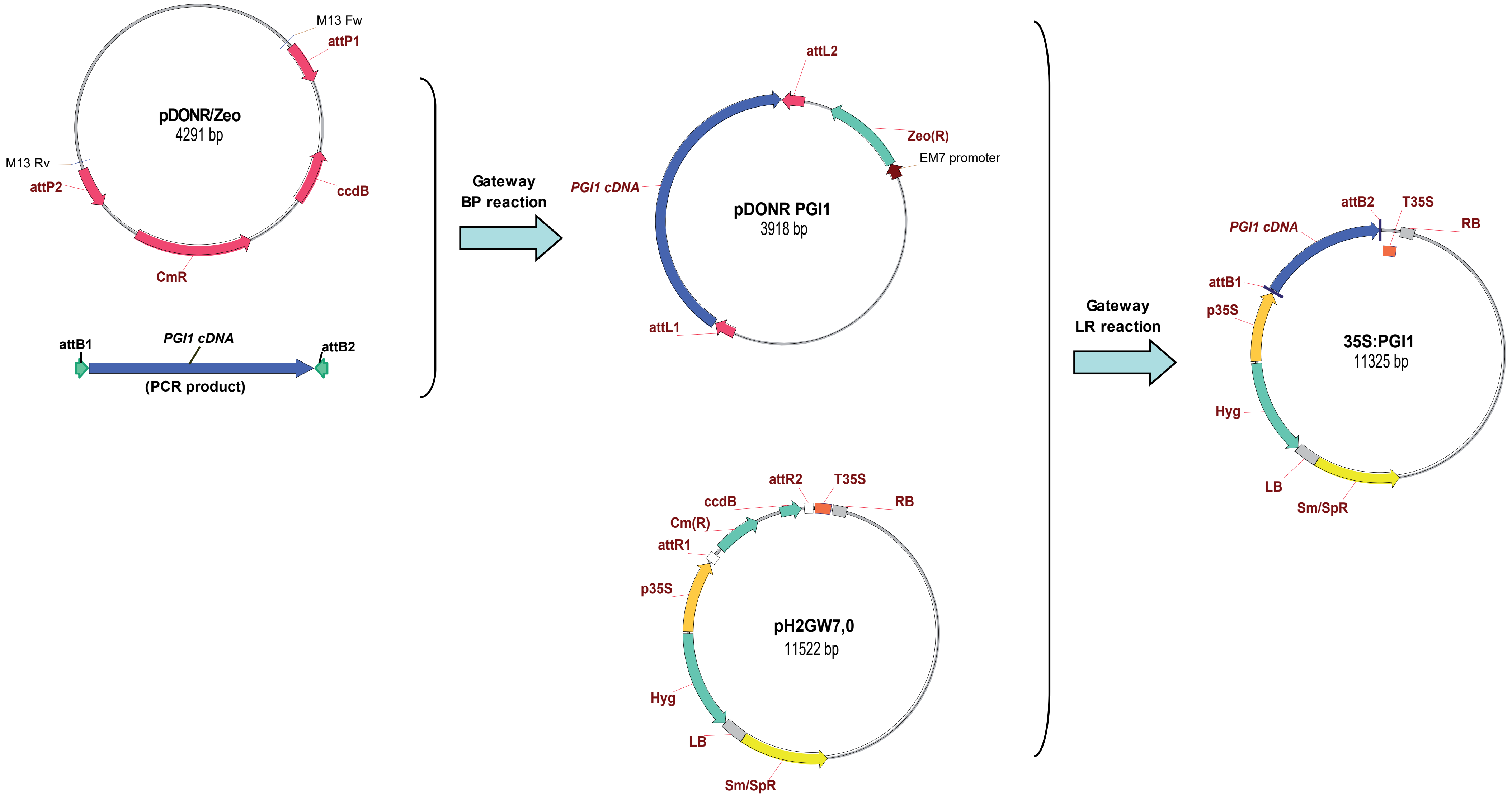

Supplemental Figure S5: Stages in the construction of the *35S:PGI1, 35S:GPT2, 35S:GPT2-GUS, promGPT2:GPT2-GUS, promGPT2:GUS, promAthspr:GPT2* and *promAthspr:GUS* plasmids. Plasmid constructs were produced using Gateway technology and confirmed by sequencing. Primers used for PCR amplification of complete *PGI1* and *GPT2* cDNAs obtained from the RIKEN Arabidopsis cDNA collection (Seki et al., 1998; Seki et al., 2002), the *Athspr* and *GPT2* promoters from genomic Arabidopsis DNA and *GUS* are listed in Supplemental Table S9.

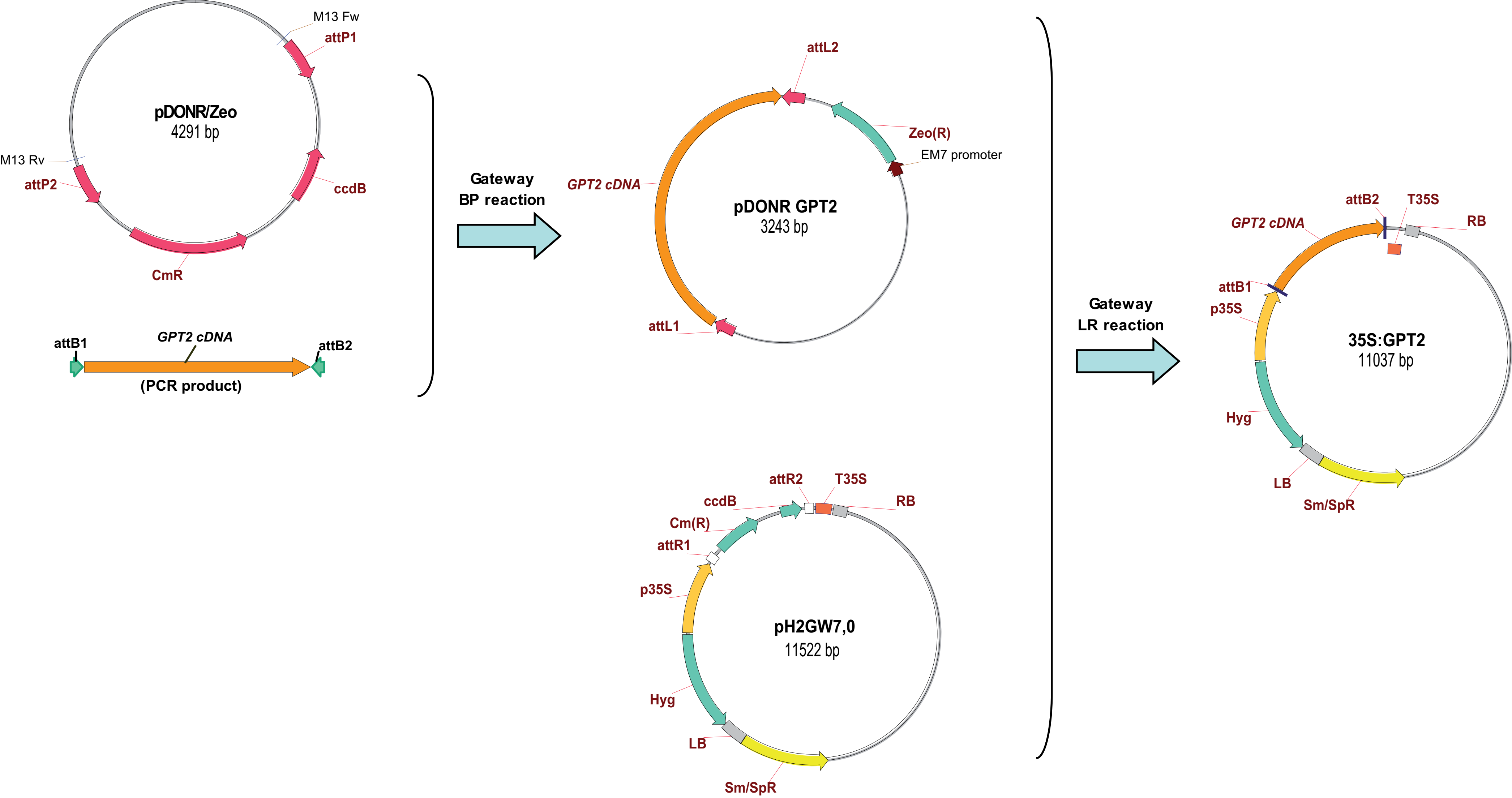
M13 Fw
attP1
pDONR/Zeo
4291 bp
M13 Rv
attP2
ccdB
CmR
attB1
GPT2 cDNA
attB2
(PCR product)
Gateway
BP reaction
attL2
Zeo(R)
EM7 promoter
GPT2 cDNA
pDONR GPT2
3243 bp
attL1
ccdB
attR2
T35S
RB
Cm(R)
attR1
p35S
pH2GW7,0
11522 bp
Hyg
LB
Sm/SpR
Gateway
LR reaction
GPT2 cDNA
attB2
T35S
RB
attB1
p35S
35S:GPT2
11037 bp
Hyg
LB
Sm/SpR

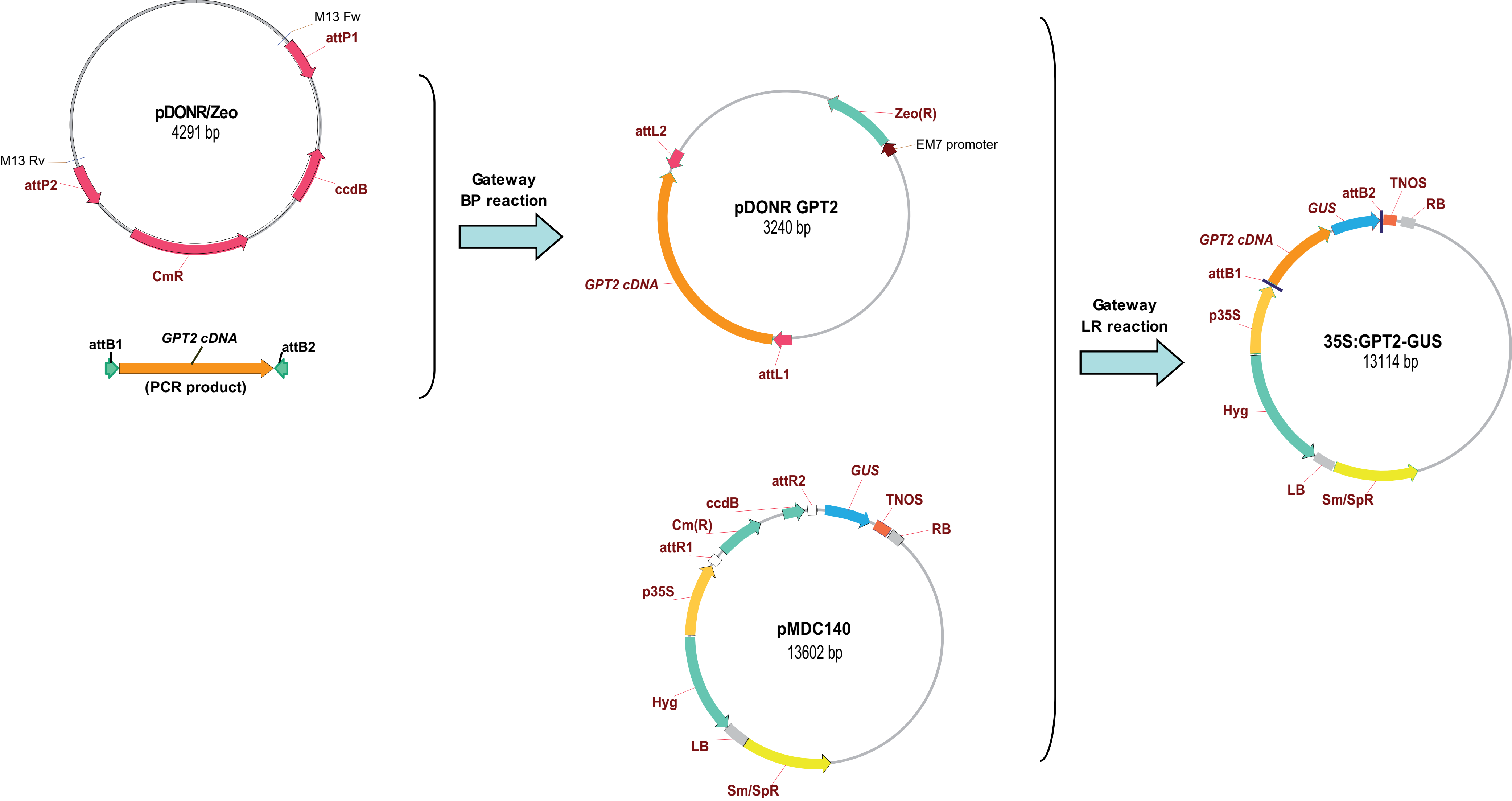

pDONR/Zeo
4291 bp
M13 Fw
attP1
M13 Rv
attP2
ccdB
CmR
attB1
GPT2 cDNA
attB2
(PCR product)
Gateway
BP reaction
pDONR GPT2
3240 bp
Zeo(R)
EM7 promoter
attL2
GPT2 cDNA
attL1
pMDC140
13602 bp
attR2
GUS
TNOS
RB
ccdB
Cm(R)
attR1
p35S
Hyg
LB
Sm/SpR
Gateway
LR reaction
35S:GPT2-GUS
13114 bp
attB2
TNOS
GUS
RB
GPT2 cDNA
attB1
p35S
Hyg
LB
Sm/SpR

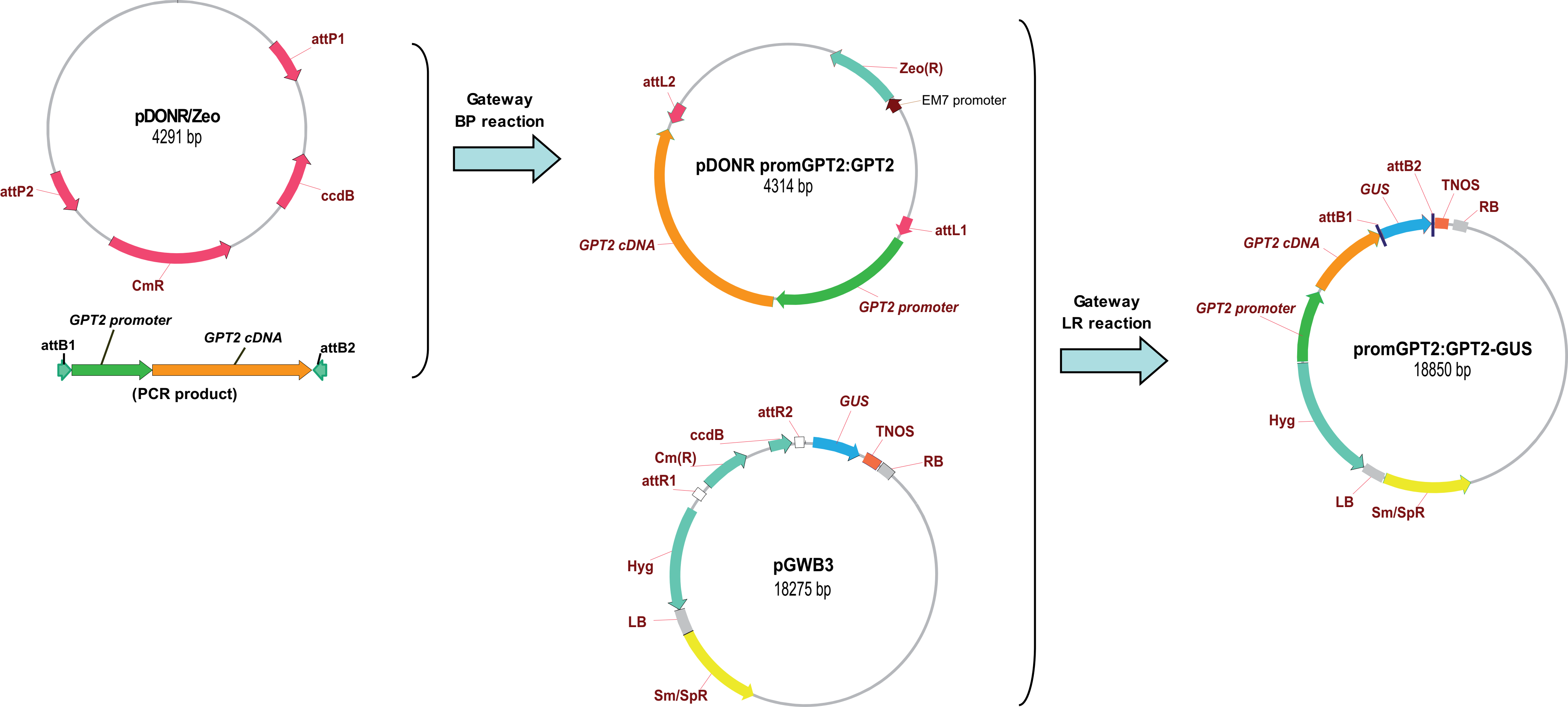

pDONR/Zeo
4291 bp
attP1
attP2
ccdB
CmR
GPT2 promoter
GPT2 cDNA
attB1
attB2
(PCR product)
Gateway
BP reaction
pDONR promGPT2:GPT2
4314 bp
attL2
Zeo(R)
EM7 promoter
attL1
GPT2 cDNA
GPT2 promoter
pGWB3
18275 bp
attR2
GUS
ccdB
TNOS
Cm(R)
RB
attR1
Hyg
LB
Sm/SpR
Gateway
LR reaction
promGPT2:GPT2-GUS
18850 bp
attB2
GUS
TNOS
RB
attB1
GPT2 cDNA
GPT2 promoter
Hyg
LB
Sm/SpR

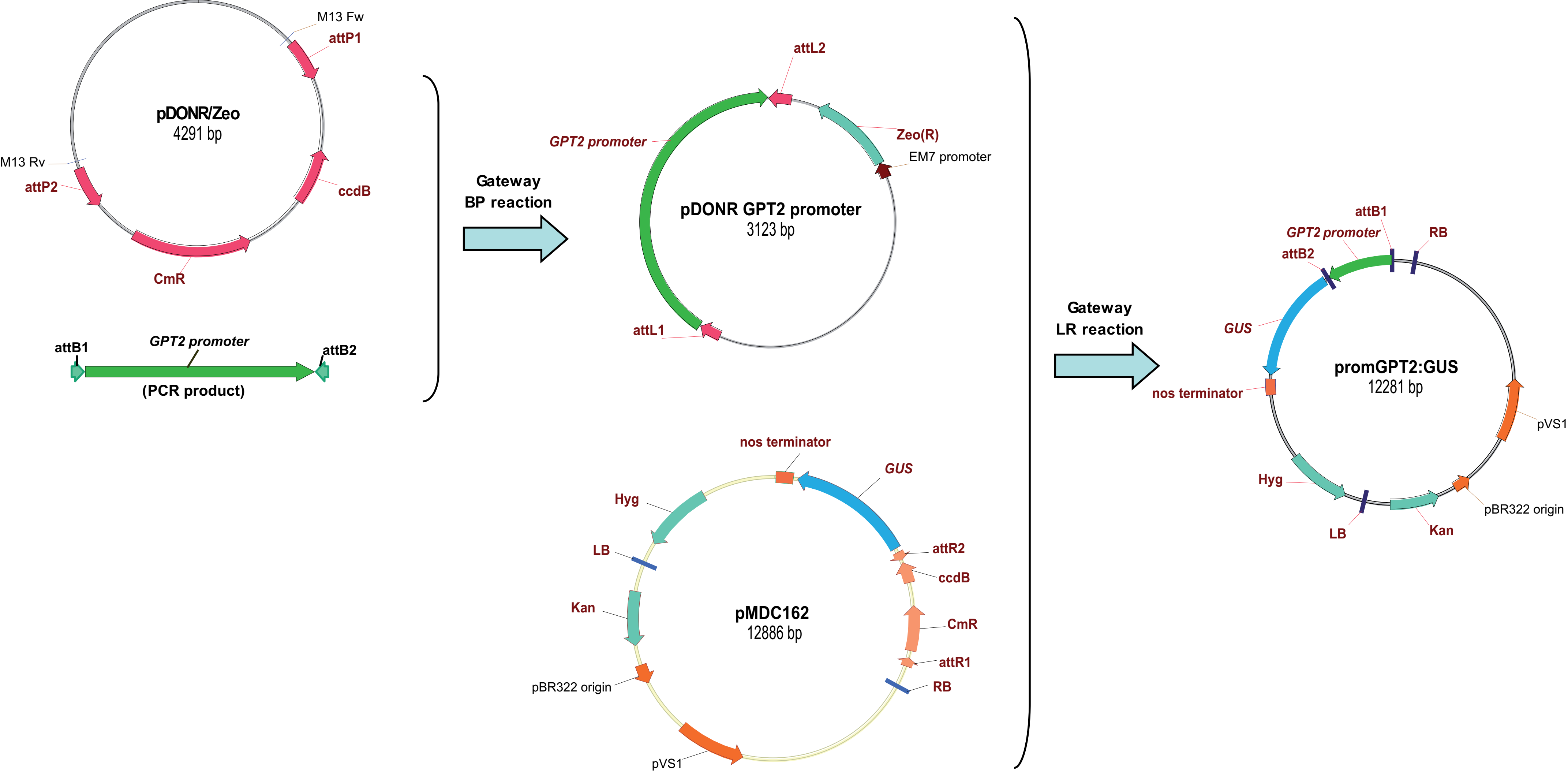

pDONR/Zeo
4291 bp
M13 Fw
attP1
M13 Rv
attP2
ccdB
CmR
attB1
GPT2 promoter
attB2
(PCR product)
Gateway
BP reaction
pDONR GPT2 promoter
3123 bp
attL2
GPT2 promoter
Zeo(R)
EM7 promoter
attL1
pMDC162
12886 bp
nos terminator
GUS
Hyg
LB
Kan
attR2
ccdB
CmR
attR1
RB
pBR322 origin
pVS1
Gateway
LR reaction
promGPT2:GUS
12281 bp
attB1
GPT2 promoter
RB
attB2
GUS
nos terminator
pVS1
Hyg
pBR322 origin
LB
Kan

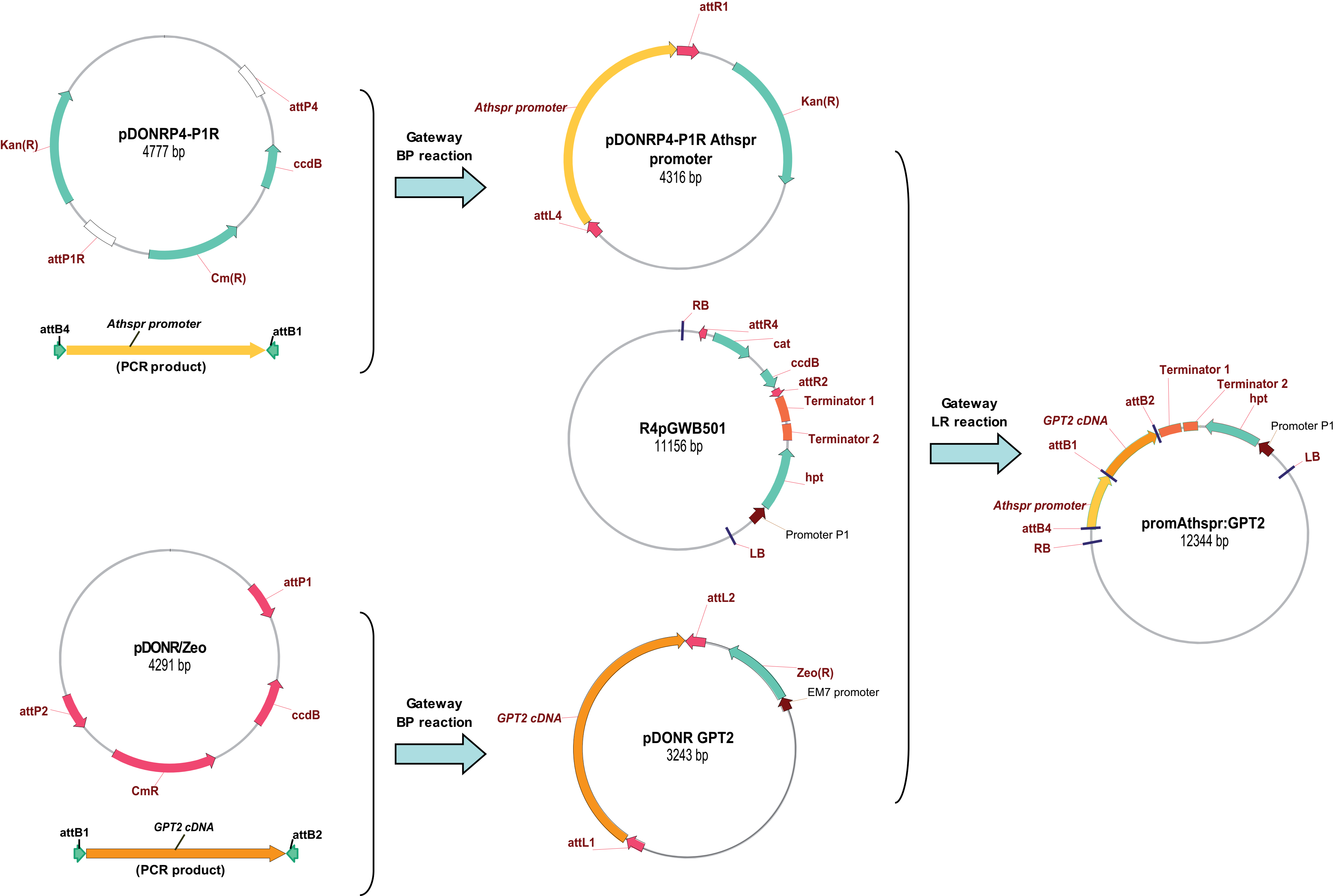
pDONRP4-P1R
4777 bp
attP4
Kan(R)
ccdB
attP1R
Cm(R)
attB4
Athspr promoter
attB1
(PCR product)
Gateway
BP reaction
pDONRP4-P1R Athspr promoter
4316 bp
attR1
Athspr promoter
Kan(R)
attL4
R4pGWB501
11156 bp
RB
attR4
cat
ccdB
attR2
Terminator 1
Terminator 2
hpt
Promoter P1
LB
Gateway
LR reaction
promAthspr:GPT2
12344 bp
Terminator 1
Terminator 2
hpt
attB2
GPT2 cDNA
Promoter P1
attB1
LB
Athspr promoter
attB4
RB
pDONR/Zeo
4291 bp
attP1
attP2
ccdB
CmR
attB1
GPT2 cDNA
attB2
(PCR product)
Gateway
BP reaction
pDONR GPT2
3243 bp
attL2
Zeo(R)
EM7 promoter
GPT2 cDNA
attL1

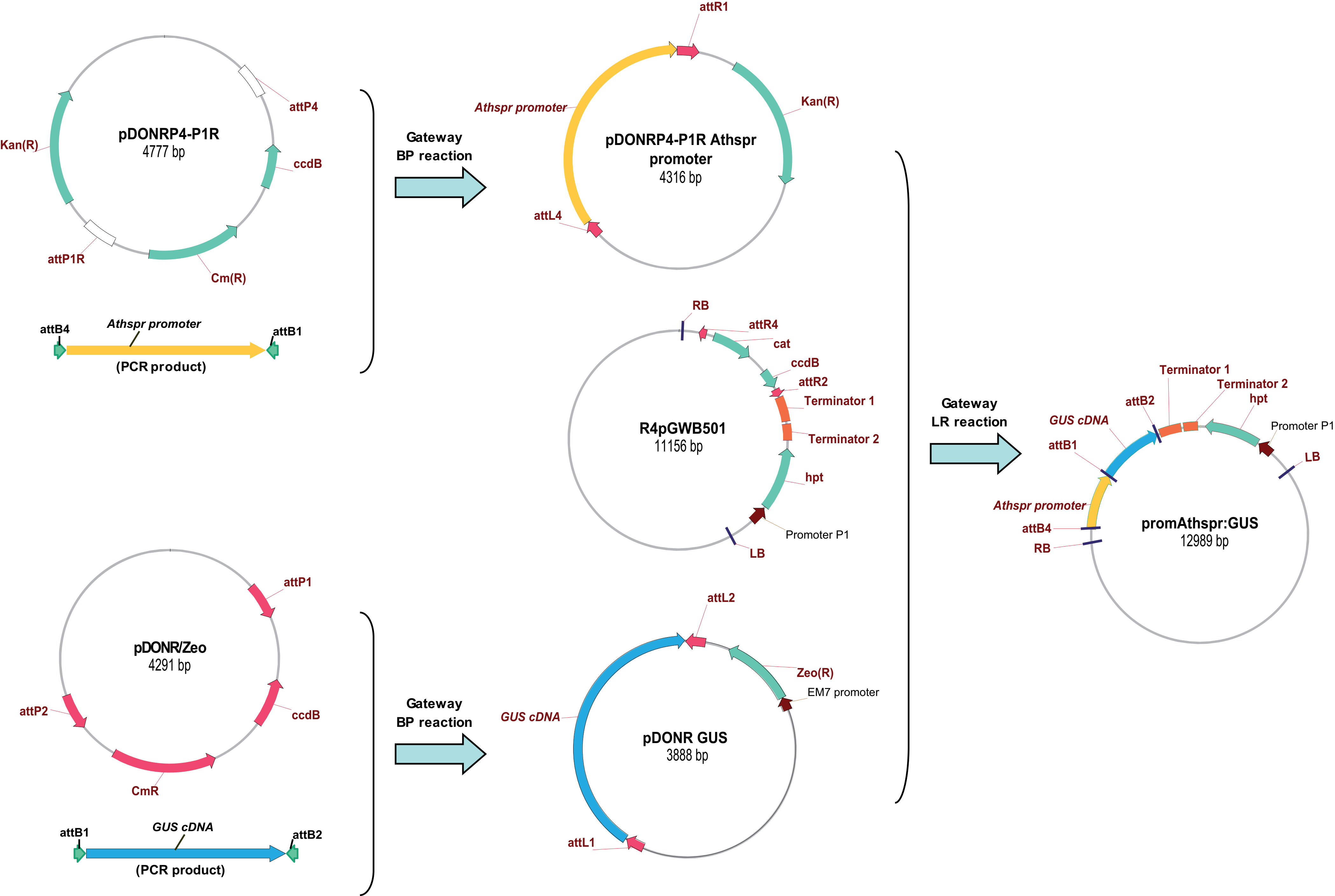

pDONRP4-P1R
4777 bp
attP4
Kan(R)
ccdB
attP1R
Cm(R)
attB4
Athspr promoter
attB1
(PCR product)
Gateway
BP reaction
attR1
Athspr promoter
Kan(R)
pDONRP4-P1R Athspr promoter
4316 bp
attL4
RB
attR4
cat
ccdB
attR2
Terminator 1
R4pGWB501
11156 bp
Terminator 2
hpt
Promoter P1
LB
Gateway
LR reaction
Terminator 1
Terminator 2
hpt
attB2
GUS cDNA
Promoter P1
attB1
LB
Athspr promoter
attB4
promAthspr:GUS
12989 bp
RB
pDONR/Zeo
4291 bp
attP1
attP2
ccdB
CmR
attB1
GUS cDNA
attB2
(PCR product)
Gateway
BP reaction
attL2
Zeo(R)
EM7 promoter
GUS cDNA
pDONR GUS
3888 bp
attL1

Supplemental Table S9. Primers used for PCR amplification of *PGI1* and *GPT2* cDNAs, *GUS* and the *Athspr* and *GPT2* promoters. Primer sequences for attB sites (see **Supplemental Figure S5**) are indicated in bold.

**Primer**	**Sequence**
**PGI1**	
attB1 *PGI1*	5'-**ggggacaagtttgtacaaaaaagcaggctta**atggcctctctctcaggc -3'
attB2 *PGI1*	5'-**ggggaccactttgtacaagaaagctgggta**ttatgcgtacaggtcatccac-3'
**GPT2**	
attB1 *GPT2*	5'**ggggacaagtttgtacaaaaaagcaggctta**atgctttcttcaatcaaaccatc-3'
attB2 *GPT2*	5'-**ggggaccactttgtacaagaaagctgggta**tcactgcttcgcctgtgag-3'
**promAthspr**	
attB4 *ATHSPR* promoter	5'-**ggggacaactttgtatagaaaagttgct**cgctctttgagttctgtagttc-3'
attB1 *ATHSPR* promoter	5'-**ggggactgcttttttgtacaaacttgc**cacaaaaccacccaccttaaatc-3'
**promGPT2**	
attB4 *GPT2* promoter	5'-**ggggacaactttgtatagaaaagttgct**gccattactttggaaaaggtcc-3'
attB1 *GPT2* promoter	5'-**ggggactgcttttttgtacaaacttgc**gtgctttttatggctaattgatg-3'
**GUS**	
attB1 *GUS*	5'-**ggggacaagtttgtacaaaaaagcaggctta**atgttacgtcctgtagaaaccc-3'
attB2 *GUS*	5'-**ggggaccactttgtacaagaaagctgggta**tcattgtttgcctccctgctg-3'

Supplemental Table S10. Primers used in RT-qPCR

Gene		Sequence
EF-1 alfa *At1g07940*	Forward	TTCGTCTCCCACTTCAGGAT
	Reverse	GGAGCAAAGGTCACAACCAT
GPT2 *At1g61800*	Forward	TTAGACCAGATTTCGCCGTTA
	Reverse	GTTGAATCGGGGTATGGAAA
GUS	Forward	GTAATTATGCGGGCAACGTC
	Reverse	TAATGAGTGACCGCATCGAA

REFERENCES TO SUPPLEMENTAL DATA

Ameztoy K, Sánchez-López ÁM, Muñoz FJ, Bahaji A, Almagro G, Baroja-Fernández E, Gámez-Arcas S, De Diego N, Doležal K, Novák O, et al (2021) Proteostatic regulation of MEP and shikimate pathways by redox-activated photosynthesis signaling in plants exposed to small fungal volatiles. Front Plant Sci doi: 10.3389/fpls.2021.637976

Seki M, Carninci P, Nishiyama Y, Hayashizaki Y, Shinozaki K (1998) High-efficiency cloning of Arabidopsis full-length cDNA by biotinylated CAP trapper. Plant J **15**: 707–720

Seki M, Narusaka M, Kamiya A, Ishida J, Satou M, Sakurai T, Nakajima M, Enju A, Akiyama K, Oono Y, et al (2002) Functional annotation of a full-length Arabidopsis cDNA collection. Science **296**: 141–145
